# Concentration‐Dependent Seeding as a Strategy for Fabrication of Densely Packed Surface‐Mounted Metal–Organic Frameworks (SURMOF) Layers

**DOI:** 10.1002/chem.202000594

**Published:** 2020-04-06

**Authors:** Qiang Li, Joshua Gies, Xiu‐Jun Yu, Yu Gu, Andreas Terfort, Martin Kind

**Affiliations:** ^1^ Institute of Inorganic and Analytical Chemistry University of Frankfurt Max-von-Laue-Strasse 7 60438 Frankfurt Germany; ^2^ Beijing Advanced Innovation Center for, Soft Matter Science and Engineering Beijing University of Chemical Technology Beijing 100029 P.R. China

**Keywords:** layer-by-layer (LbL), metal–organic frameworks, surface chemistry, surface-mounted metal–organic frameworks (SURMOFs), Volmer–Weber growth

## Abstract

The layer‐by‐layer (LbL) method is a well‐established method for the growth of surface‐attached metal–organic frameworks (SURMOFs). Various experimental parameters, such as surface functionalization or temperature, have been identified as essential in the past. In this study, inspired by these recent insights regarding the LbL SURMOF growth mechanism, the impact of reactant solutions concentration on LbL growth of the Cu_2_(F_4_bdc)_2_(dabco) SURMOF (F_4_bdc^2−^=tetrafluorobenzene‐1,4‐dicarboxylate and dabco=1,4‐diazabicyclo‐[2.2.2]octane) in situ by using quartz‐crystal microbalance and ex situ with a combination of spectroscopic, diffraction and microscopy techniques was investigated. It was found that number, size, and morphology of MOF crystallites are strongly influenced by the reagent concentration. By adjusting the interplay of nucleation and growth, we were able to produce densely packed, yet thin films, which are highly desired for a variety of SURMOF applications.

## Graphical abstract

**Insights from in situ and ex situ** investigation on the concentration dependency of the layer‐by‐layer (LbL) growth of metal–organic frameworks on surfaces markedly improve control of the LbL process. Even at reduced cycle numbers, by varying reactant concentrations, densely packed yet thin films could be produced, which are highly desired for various applications.

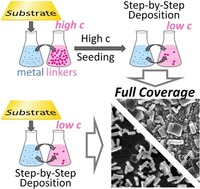

Surface‐anchored metal–organic frameworks (SURMOFs) are currently developed for applications, such as chemical sensors,^1^, ^2^, ^3^ electronic devices,^3^, ^4^, ^5^ membranes,^2^, ^6^, ^7^, ^8^ optics,^5^ or photovoltaics.^9^, ^10^ A crucial precondition for functional SURMOFs is the control over the growth of MOF thin films on suiting surfaces and their properties, such as composition, thickness, packing density, or orientation.^11^ Several methods to produce SURMOFs have been developed.^12^ A very popular one is the layer‐by‐layer (LbL) method, also known as liquid‐phase epitaxy, in which the substrates are sequentially exposed to solutions of the metal precursors and of the ligands, respectively, with intermediate washing steps.^13^ LbL has been used to deposit different MOF types, for example, Zn_3_(btc)_2_ 14 (btc=1,3,5‐benzenetricarboxylate) and HKUST‐1,^13^, ^15^ but also more complex systems with two different organic linker species, such as Cu_2_(ndc)_2_(dabco) (ndc=1,4‐naphthalenedicarboxyate, dabco=1,4‐diazabicyclo[2.2.2]octane),^16^ Cu_2_(F_4_bdc)_2_(dabco),^17^ or Cu_2_(sdb)_2_(bipy) (sdb = 4,4’‐sulfonyldibenzoate, bipy = 4,4′‐bipyridine).^18^ The details of SURMOF formation mechanisms by using the LbL method are still under discussion,^19^ but a couple of important parameters for controlling and directing SURMOF growth, such as surface functionality,^13^, ^14^, ^15^, ^16^, ^17^ temperature,^17^, ^20^ surface energy,^18^ or surface defects^21^ have been identified and discussed in the literature. Early publications describe the LbL SURMOF growth as a Frank van der Merve process,^13^, ^14^, ^15^ but it is generally accepted today that the LbL growth is in fact a Volmer–Weber process resulting in surfaces covered by solitary crystals.^17^, ^20^, ^22^, ^23^, ^24^ Although a careful optimization of parameters permits the growth of highly oriented material, the Volmer–Weber mechanism inherently prohibits the growth of thin and closed layers. Only at higher coverages, the crystals coalesce to form a closed, but rather thick SURMOF film. Thin but closely packed SURMOF films are desirable, for example, for molecular sieving^7^, ^8^ or in sensing applications,^25^ in which a fast transport or uptake^26^ of guest molecules is desirable. Apart from this thickness issue, LbL often is rather time consuming with single immersion cycles lasting up to 90 minutes^14^, ^17^, ^18^, ^20^, ^22^, ^27^, ^28^, ^29^, ^30^ and standard cycle numbers of 40 and more.^15^, ^16^, ^17^, ^18^, ^24^, ^27^, ^28^, ^29^, ^31^, ^32^, ^33^, ^34^, ^35^ Moreover, with each LbL cycle, a certain amount of material has to be discarded, rendering this method rather consumptive, especially in the context of experiments, in which non‐commercially available organic linker molecules are used. For these reasons, in the present study, we address parameters that allow formation of thin but closely packed SURMOF layers being produced by using the LbL technique in a reasonable time and with a minimized material usage. Based on recent studies of the LbL SURMOF growth mechanism,^17^, ^20^, ^22^, ^23^ which suggest that reagent concentration might play a role in the deposition process, we wanted to systematically vary this parameter. We chose the established pillared‐layer MOF Cu_2_(F_4_bdc)_2_(dabco)^36^ (linker: F_4_bdc^2−^=tetrafluorobenzene‐1,4‐dicarboxylate, pillar: dabco=1,4‐diazabicyclo‐[2.2.2]octane), because this system sensitively reacts to changes of LbL parameters.^17^, ^37^ Moreover, the infrared spectrum of the F_4_bdc^2−^ linker exhibits shifts in the carboxylate bands that allow for a more accurate distinction of the involved species in the spectroscopic data.^17^ Following the established protocols,^1^, ^13^, ^14^, ^15^, ^16^, ^17^, ^18^, ^20^, ^22^, ^23^, ^24^, ^30^, ^38^ we carried out LbL experiments on gold‐covered quartz crystal microbalance (QCM) substrates that were functionalized with pyridyl‐terminated self‐assembled monolayers.^39^ To understand the processes in the earlier stages of SURMOF growth (before meaningful features are overgrown), we limited the number of deposition cycles to 20. MOF growth characteristics were observed by QCM, X‐ray diffraction (XRD), reflection/absorption infrared spectroscopy (IRRAS), scanning electron microscopy (SEM), and atomic force microscopy (AFM; see the Supporting Information for experimental details).

As a general remark, the results of the characterization of all samples by IR spectroscopy and X‐ray diffraction are in line with formation of the desired MOF system. In some of the IR spectra, there are hints on amorphous or even non‐MOF material, as indicated by, for example, bands above 1700 cm^−1^ (compare Figure 1, left side). However, the amount of this material is much lower than ten percent. Varying the concentrations of the pillar and linker molecules and the copper source, however, resulted in different amounts of deposited material as was indicated by the QCM results and a remarkable variety of crystallite shapes, sizes (compare the respective micrographs in Figure 2 and in the Supporting Information), surface area densities (see Figure S42 in the Supporting Information) and roughness value (see Figure S44).

**Figure 1:**
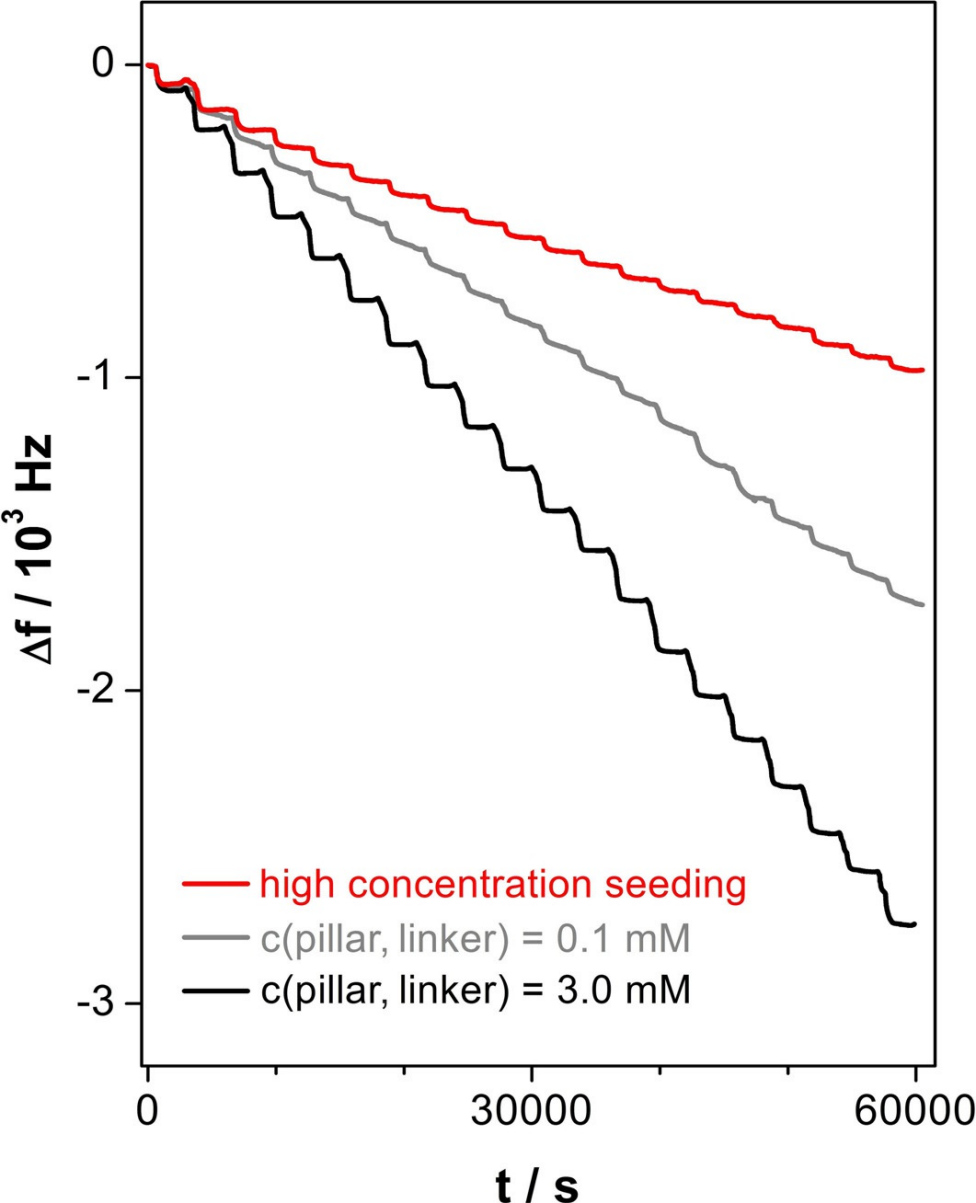
QCM LbL Cu_2_(F_4_bdc)_2_(dabco) deposition curves (20 cycles) at 1 mm Cu^2+^. High‐concentration seeding: c(pillar, linker)=3 mm in the first cycle and 0.1 mm in the following cycles.

**Figure 2:**
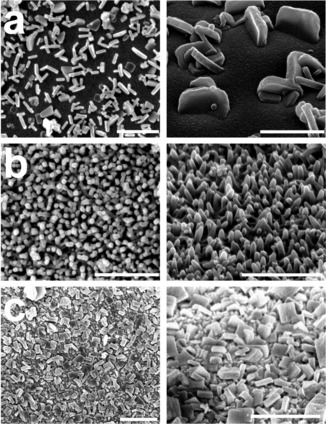
SEM images of Cu_2_(F_4_bdc)_2_(dabco) SURMOFs after 20 LbL deposition cycles at 1 mm Cu^2+^. Left: top views, right: tilted views. (a) All cycles with c(pillar, linker)=0.1 mm; (b) all cycles with c(pillar, linker)=3 mm; (c) high‐concentration seeding (first cycle c(pillar, linker)=3 mm, all other cycles c(pillar, linker)=0.1 mm). All scale bars=1 μm.

To efficiently explore the concentration range, we went to the solubility limits of the respective reagents (pillar, linker: 3 mm, Cu^2+^: 20 mm). As was demonstrated by the in situ QCM measurements, under these conditions more than five times of the material became deposited compared to the standard conditions (pillar, linker: 0.1 mm, Cu^2+^: 1 mm; see Figure S35 in the Supporting Information);^17^ but the QCM sensorgram of this experiment does not show regular LbL steps^16^, ^29^, ^31^, ^32^, ^33^, ^35^, ^40^, ^41^ (Figures S19 and S20 in the Supporting Information). Morphology and crystallinity of the deposit were unsatisfactory (compare SEM data in Figure S31). Surprisingly, both diffraction (Figure S33) and spectroscopic (Figure S34) data nevertheless suggest the formation of the desired MOF system. To elucidate at which concentration the transition of the ordered growth of crystallites into the observed disorder takes place, we varied the concentration of the ligand mixture (pillar, linker), and the one of the Cu^2+^ independently from each other. In a first LbL series, we kept the Cu^2+^ concentration at 1 mm and applied pillar, linker concentrations of 0.1 to 3 mm. All resulting QCM curves are on first sight compliant with the assumption of a regular LbL process (black and gray curves in Figure 1, Figures S3–S12 in the Supporting Information); the amount of deposited material slightly increased with increasing pillar, linker concentration (Figure S35). Note, though, that the amount of deposited material per surface area is markedly higher than would be expected from the previously favored liquid‐phase epitaxy model (addition of one MOF layer per cycle), by about one order of magnitude. We interpret this outcome as another indication that this model is not applicable. Note that also other studies on LbL growth of pillar‐layer SURMOFs report deposition of more material than expected.^42^ Nevertheless, at the lowest concentrations (pillar, linker: both 0.1 mm, Cu^2+^: 1 mm) our previous results^17^ could be reproduced (see SEM data in Figures 2 a and S23, gray XRD and IR curves in Figure 3, as well as the XRD and IR data in Figures S33 and S34, respectively). The analysis of the XRD data of these samples, based on a bimodal model (assuming that all crystallites are either (100) or (001) oriented)^17^ suggests that the orientational quality increases with increasing pillar, linker concentration (Figure S40). But under these conditions, a discrepancy with the orientational data obtained by analysis of the IR spectra arises (also Figure S40). The SEM images reveal the likely reason for this: When the pillar and linker concentration is varied from 0.1 to 1 mm, the deposited crystals become smaller, stubbier and more numerous, and apparently the growth perpendicular to the (001) direction became less dominant (Figures S24 and S25 in the Supporting Information). At a pillar, linker concentration of 1.5 mm, the crystals change from a more plate‐like into a conical form (Figures S26 and S27). Orientations different from (100) and (001) appear, many of which are not detectable by XRD due to their high index nature, which leads to the discrepancy between the IR and the XRD results. We interpret the presence of such spiky structures to be correlated with a change in deposition mechanism, which is due to an increased supersaturation that induces a diffusion‐limited adhesive growth rather than a birth‐and‐spread growth (Figure 4, center).^19^ As additional orientations appear at higher pillar, linker concentrations, the simple binary model to evaluate the proportion of (001) orientation is not applicable anymore. To take this into account, we derived a formula to obtain an average tilt angle from the IR data (see the Supporting Information). This tilt angle is indeed affected by the pillar, linker concentration (see Figure S41), but the concentration effect on the orientation is not as high as the one of temperature.^17^

**Figure 3:**
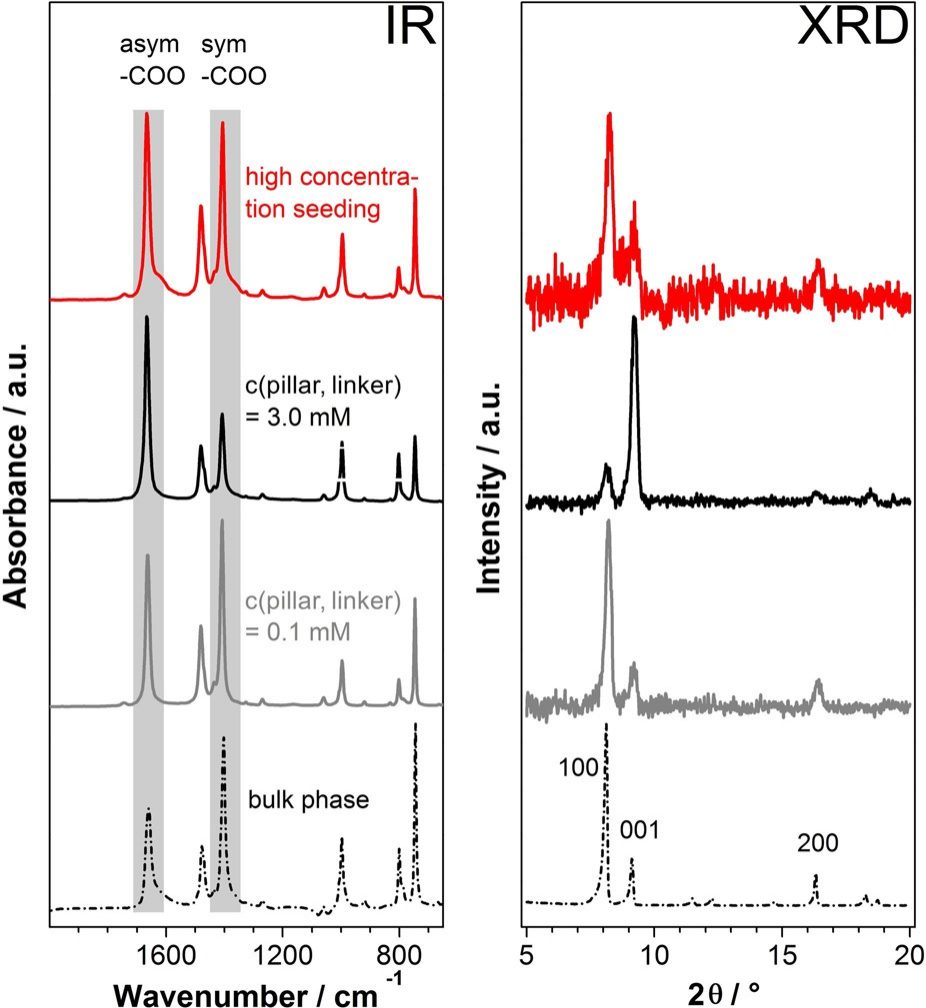
Infrared spectra (left) and X‐ray diffraction patterns (right) of Cu_2_(F_4_bdc)_2_(dabco) SURMOFs after 20 LbL deposition cycles at 1 mm Cu^2+^, compared to bulk phase MOF data. High‐concentration seeding: c(pillar, linker)=3 mm in the first cycle and 0.1 mm in the following cycles.

**Figure 4:**
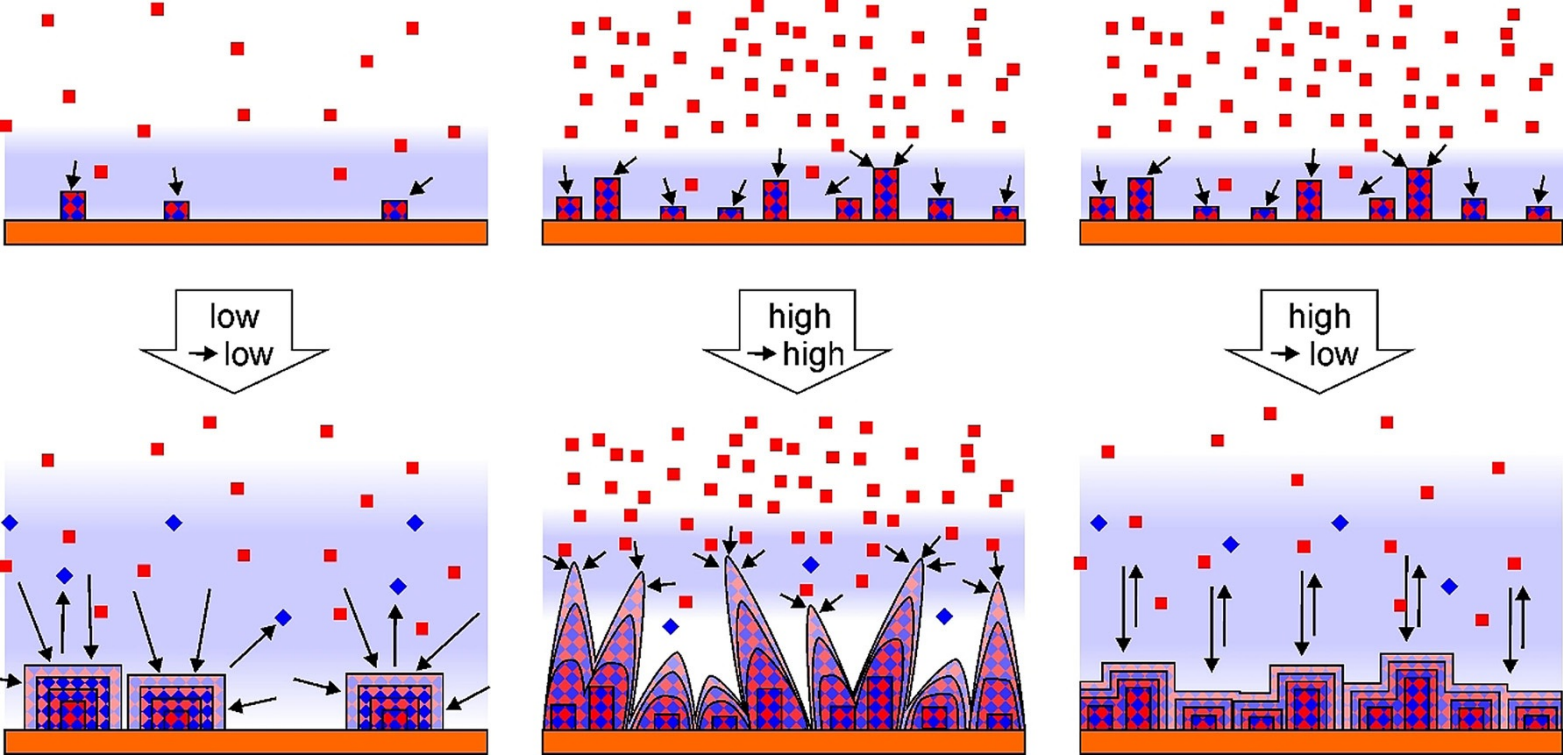
Scheme of the mechanism of the concentration‐dependent SURMOF deposition. Left: At low concentrations, equilibria are established within a relatively wide diffusion layer (light blue background). This results in a low density of nuclei, but in very defined crystallites at later stages. Closed layers are only obtained when the size of the crystals exceeds their average distance. Middle: At high concentrations, nucleation is very efficient, but due to a very narrow diffusion zone the crystals become dendritic. Right: By combining the processes, dense layers of nuclei can be obtained, which become closely packed after only few deposition steps. The crystals are well defined due to the equilibrium formation, but growth is slow due to 2D transport. Other phenomena, such as Ostwald ripening, material storage, and the orientational disorder are omitted for clarity.

To examine the impact of the Cu^2+^ concentration on SURMOF formation, we also performed LbL experiments at constant pillar, linker concentration and varying Cu^2+^ concentrations. At a Cu^2+^ concentration of 3 mm, the QCM sensorgrams became atypical, as can be seen in the higher cycle regime in Figure S13, in which the step height continuously decreases and the steps almost vanish. At c(pillar, linker)=0.1 mm and c(Cu^2+^)=20 mm, during the exposure to copper solution, even a loss of material was observed (Figures S17 and S18 in the Supporting Information), which cannot be explained within the liquid‐phase epitaxy model. Instead, this finding is an indication of dissolving reactants and a process of crystallization of the MOF out of a solution that contains all reactants at the same time (Figure 4), a mechanism that has already been proposed for other SURMOF systems.^18^, ^24^ Although both the XRD and the IR data of these samples do not show a significant deviation from the classical c(Cu^2+^)=1 mm, c(pillar, linker)=0.1 mm experiment (see Figures S33 and S34 in the Supporting Information), the SEM images (Figures S28 and S30) show a considerable twinning of the deposited crystallites at the higher Cu^2+^ concentrations. Although at 3 mm Cu^2+^, the amount of material determined by QCM was similar to the 1 mm Cu^2+^ case, twice as much of the SURMOF material became deposited at 20 mm Cu^2+^ concentration (Figure S35). As was shown by the micrographs, this is caused by a significantly higher density of crystallites at the latter case, although the size of the crystals was similar for both concentrations (compare Figures S28 and S30). Although no spikes were found in these cases, for 3 mm Cu^2+^ and the highest pillar, linker concentration of 3 mm, a pronounced formation of spikes was observed (Figure S29).

Obviously, this strong dependence of the number, size, and morphology of the MOF crystallites on reactant concentrations is a consequence of the competition of nucleation and growth of the MOF crystallites, combined with the transition between different growth modes, that is, birth‐and‐spread versus diffusion limited growth.^19^ On the one hand, higher reactant concentrations clearly prohibit a SURMOF buildup following the liquid phase epitaxy model. On the other hand, even at the lowest concentrations at which LbL can be executed, there is lots of evidence that all reactants are present in the solution at the same time and that MOF growth actually is a (pseudo‐)equilibrium crystallization process out of this solution rather than liquid‐phase epitaxy^17^, ^22^, ^23^, ^24^ (see Figure 4). In line with this model, prolonging the deposition time does not increase the amount of deposited material (see the results of an according experiment in Figures S45–S48 in the Supporting Information).

Based on these considerations, we figured that we could achieve the deposition of a low amount of MOF material that yet form a closely packed layer on the substrate surface by making use of varying concentrations during the LbL procedure. A high reactant concentration during early LbL cycles provides a high number of crystallization nuclei, whereas low concentrations later in the LbL procedure limits the amount of deposited MOF material (Figure 4, right column). We kept the Cu^2+^ concentration constant at 1 mm and applied c(pillar, linker)=3 mm in the first cycle and 0.1 mm in the subsequent cycles (“high‐concentration seeding”). IR and XRD (Figure 3, red curves and Figures S33 and S34) suggest that the Cu_2_(F_4_bdc)_2_(dabco) MOF was formed in this experiment. SEM reveals that after high‐concentration seeding, the substrate is completely covered with crystallites smaller and more numerous than after LbL with constant reactant concentrations (compare Figure 2 c with a, also Figures S32 with S23 in the Supporting Information). We also found the desired regular behavior of the QCM sensorgram—with a slight decrease in the frequency steps at higher cycles (red curve in Figure 1, Figures S21 and S22). As was assumed, the quantity of material deposited by high‐concentration seeding turned out to be lower than in the case of the conventional LbL experiments at pillar, linker concentrations of 3.0 mm and even 0.1 mm (Figures 1 and S35 in the Supporting Information). From a statistics of crystallites formed during the LbL procedure (Figure S42), we estimate the number of initial nuclei to be higher by a factor of approximately 40 in comparison to the c(pillar, linker)=0.1 mm case. The large initial number of nuclei in the high‐concentration seeding experiment is the reason for both the great number and the small size of the crystallites formed and for the lower amount of deposited material, because the coalescence of the crystallites limits their respective growth during the LbL experiment (transition from 3D to 2D growth). Note that in the high‐concentration seeding case, the crystallites show a slightly broader size distribution and are less oriented, but are still plate‐like (Figure 2 c) as in the standard concentration case (Figure 2 a) and in contrast to the spike‐like morphology at high pillar, linker concentration (Figure 2 b). Moreover, the high concentration seeding sample exhibited a relatively low roughness compared to all other samples (see Figure S44). This appears to be in line with the fact that the crystallites produced by high concentration seeding are markedly smaller in comparison to the ones from all other LbL experiments (compare Figure S43; note that these data should be taken with a grain of salt due to the high variation of crystallite shapes that limits the comparability between the samples).

In conclusion, by systematically varying the reactant concentrations in LbL SURMOF deposition experiments, we found a strong impact on the growth mechanism resulting in different density, size, and morphology of the MOF crystallites, which adds on top of the Volmer–Weber growth mechanism. Consequently, we employed this concentration dependence as a new tool to control the SURMOF LbL growth that joins surface functionality,^13^, ^14^, ^15^, ^16^, ^17^ temperature,^17^, ^20^ and surface energy.^18^ We found that exploiting the competition between seeding and growth in SURMOF formation^19^ by high concentration seeding during the first step of the LbL procedure opens the possibility for the fabrication of closely packed SURMOFs yet at low deposited amounts and at a markedly lower number of LbL cycles than otherwise necessary to fully cover the substrate surface. This new approach further expands the control of the growth of tailor‐made SURMOF systems for specific applications and in addition offers valuable time and material savings in the SURMOF production process.

## Conflict of interest

The authors declare no conflict of interest.

## Supporting information

As a service to our authors and readers, this journal provides supporting information supplied by the authors. Such materials are peer reviewed and may be re‐organized for online delivery, but are not copy‐edited or typeset. Technical support issues arising from supporting information (other than missing files) should be addressed to the authors.

Supplementary
