# Supplementary material for: Concentration‐Dependent Seeding as a Strategy for Fabrication of Densely Packed Surface‐Mounted Metal–Organic Frameworks (SURMOF) Layers
Source: Chemistry. 2020 Apr 6;26(23):5185–9. doi: 10.1002/chem.202000594 (PMC7217006; doi:10.1002/chem.202000594)
Supplement: Supplementary file 1 — Supplementary [file CHEM-26-5185-s001.pdf]

Chemistry–A European Journal

Supporting Information

Concentration-Dependent Seeding as a Strategy for Fabrication of Densely Packed Surface-Mounted Metal–Organic Frameworks (SURMOF) Layers

Qiang Li,[a] Joshua Gies,[a] Xiu-Jun Yu,[a] Yu Gu,*[b] Andreas Terfort,*[a] and Martin Kind*[a]

Index

Sketch of the $Cu_2(F_4bdc)_2$(dabco) crystal structure 2

Experimental Details 3

Materials 3

SURMOF preparation 3

Infrared Spectroscopy 4

X-Ray Diffraction 4

Microscopy 4

Details on the Calculation of Infrared Spectra 4

Bulk Infrared Spectra of Pillar, Linker and $Cu_2(F_4bdc)_2$(dabco) MOF 5

Band Assignment of Infrared Spectra 6

Layer-by-Layer Experiments: QCM Curves 7

Layer-by-Layer Experiments: SEM Images 17

X-ray Diffraction Data of $Cu_2(F_4bdc)_2$(dabco) Powder and SURMOFs 23

Infrared Spectra of $Cu_2(F_4bdc)_2$(dabco) Bulk and SURMOF Samples 24

QCM: LbL Relative Mass Changes 25

Mass Changes after 20 LbL Cycles 25

Full Cycle Mass Changes 26

Mass Changes during Rinsing with Cu Solution 27

Mass Changes during Rinsing with Pillar/Linker Solution 29

Orientation of the SURMOFs 30

Proportion of (001) Orientation 30

Average tilt angle from IR data 31

Surface density and sizes of SURMOF crystallites 32

Surface density 32

Approximate crystallite sizes 33

Roughness of SURMOFs 34

LbL experiment with increased durations of pumping cycles 35

Evaluation Formula to Obtain the Average Tilt Angle from IR Intensities 37

References 41

Sketch of the $Cu_2(F_4bdc)_2(dabco)$ crystal structure

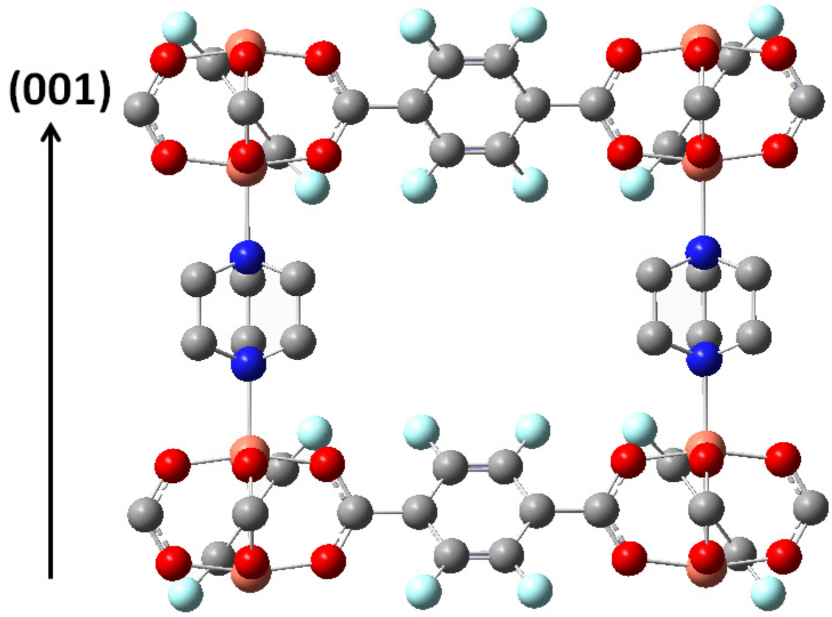

Figure S1. Sketch of the $Cu_2(F_4bdc)_2(dabco)$ MOF structure. Atom colors: gray - carbon, dark blue - nitrogen, red - oxygen, light blue - fluorine, orange - copper. H atoms have been omitted for clarity. Both F_4bdc^{2-} linkers as well as the dabco pillars are rotationally disordered, which has been omitted for clarity as well. The arrow indicates the crystallographic (001) direction. Both directions (100) and (010) perpendicular to (001) are equivalent. Structural information adopted from ref.[1]

Experimental Details
Materials

Reagents: copper(II) acetate monohydrate ($Cu(CH_3COO)_2 \cdot H_2O$) (99% purity), tetrafluoroterephthalic acid (H_2F_4bdc) (97% purity), 1,4-diazabicyclo(2.2.2)octane (dabco) (98% purity), and absolute ethanol (99.99% purity) used in the experiments were purchased from Carl Roth GmbH + Co. KG, Germany, Apollo Scientific, UK, Merck Schuchardt OHG, Germany, and Fisher Scientific U.K. Limited, CA, respectively. The commercially purchased reagents were used as received without further purification. For functionalization of the substrates with a self-assembled monolayer, [4-[4-(4-pyridyl)phenyl]phenyl]methanethiol (PPP1) was synthesized as described in literature.[2] $Cu_2(F_4bdc)_2$(dabco) bulk MOF material was synthesized according to literature.[3]

SURMOF substrates: in this work, SURMOFs were grown on QCM probes (5 MHz, 1" diameter, AT-cut quartz crystal wafers with Ti/Au circular electrodes on both sides) from Stanford Research Systems Inc. Self-assembled monolayer formation experiments with dodecanethiol out of ethanolic solution resulted in a frequency change of $\Delta f \approx 50$ Hz per 1 $\mu g/cm^{-2}$ deposited material.

SURMOF preparation

Substrate functionalization by deposition of a PPP1 self-assembled monolayer (SAM): The QCM crystals were rinsed with absolute ethanol (10 mL) for 30 seconds, dried under N_2 stream for 30 seconds, followed by a 5 minutes treatment in H_2 plasma, and immersed into a 0.1 mM PPP1 solution (absolute ethanol as solvent) for 24 hours at room temperature. After rinsing with absolute ethanol and drying in a stream of N_2 for 30 seconds, the SAM-functionalized QCM crystals were used in SURMOF deposition experiments.

Layer-by-Layer (LbL) growth of $Cu_2(F_4bdc)_2$(dabco) SURMOFs on the substrates: SURMOF growth was performed in a flow cell (Stanford Research Systems Inc.) using a LbL setup with a four-channel peristaltic pump (UD-78001-80, Cole-Parmer GmbH, Germany). The frequency signals of the QCM crystals were logged using a QCM 200 system (Stanford Research Systems Inc., CA) during SURMOF growth. Absolute ethanol was used as solvent for the copper(II) acetate solution and the $H_2(F_4bdc)$/dabco solutions and as rinsing agent. For each experiment, copper(II) acetate solution, absolute ethanol, $H_2(F_4bdc)$/dabco solution, and absolute ethanol were pumped consecutively through the flow cell at a flow velocity of 200 µl/min for 400, 200, 2000, and 400 seconds, respectively. In an additional experiment, the durations of the respective steps were doubled (800, 400, 4000, and 800 seconds). LbL cycles were repeated 20 times. Concentrations of the employed solutions were systematically varied (copper(II) acetate: from 0.1 to 3.0 mM, $H_2(F_4bdc)$/dabco: from 1.0 to 20.0 mM) to study the concentration dependency of material deposition onto the QCM substrates.

All experiments were conducted at room temperature.

Infrared Spectroscopy

For spectroscopic analysis of the SURMOF coated samples, a Nicolet 6700 Fourier transform infrared (IR) spectrometer (Thermo Fisher, Germany) equipped with a with liquid nitrogen cooled narrow-band mercury cadmium telluride detector was used. The optical path was purged with dry, CO_2-free air during the measurements. The spectral range and the resolution were 650-4000 cm^{-1} and 4 cm^{-1}, respectively. Infrared reflection-absorption spectroscopy (IRRAS) of the SURMOFs was conducted using a reflection-absorption unit for measurements at an 80° angle of incidence. A self-assembled monolayer prepared from perdeuterated hexadecanethiol ($C_{16}D_{33}SH$) on gold substrate was used as a reference for the IRRAS measurements. IR spectra of bulk phase MOF material were collected with an attenuated total reflection unit using the same spectrometer.

X-Ray Diffraction

Surface X-ray diffraction (SXRD) measurements of SURMOF coated samples were performed with a theta-theta diffractometer (STOE, Germany) using Cu Kα (1.5418 Å) radiation and a linear position sensitive detector, with a step width of 0.02° between 2θ = 5° and 20°, and a scan rate of 10 s/step. Powder X-ray diffraction patterns of bulk MOF material were collected with a StadiP diffractometer (STOE, Germany) between 2θ = 2° and 70° using Cu $K\alpha_1$ (1.5406 Å) radiation.

Microscopy

Scanning electron microscopy (SEM) images were recorded with an Amray 1920 ECO SEM (SEMTech Solutions, Inc., Billerica, MA) or with a Nova NanoLab 600 SEM/FIB (FEI, OR).

Atomic force microscopy (AFM) was performed using a SOLVER PRO atomic force microscope (NT-MDT, Russia).

Details on the Calculation of Infrared Spectra

The Gaussian 09 program package[4] was used to calculate IR spectra of isolated molecules with density functional theory. The BP86 functional[5,6] and the SVP basis set[7] were used. Calculated wavenumbers were not scaled. Spectra calculations served to assign the vibrational modes and to identify the directions of their transition dipole moments.

Bulk Infrared Spectra of Pillar, Linker and $Cu_2(F_4bdc)_2$(dabco) MOF

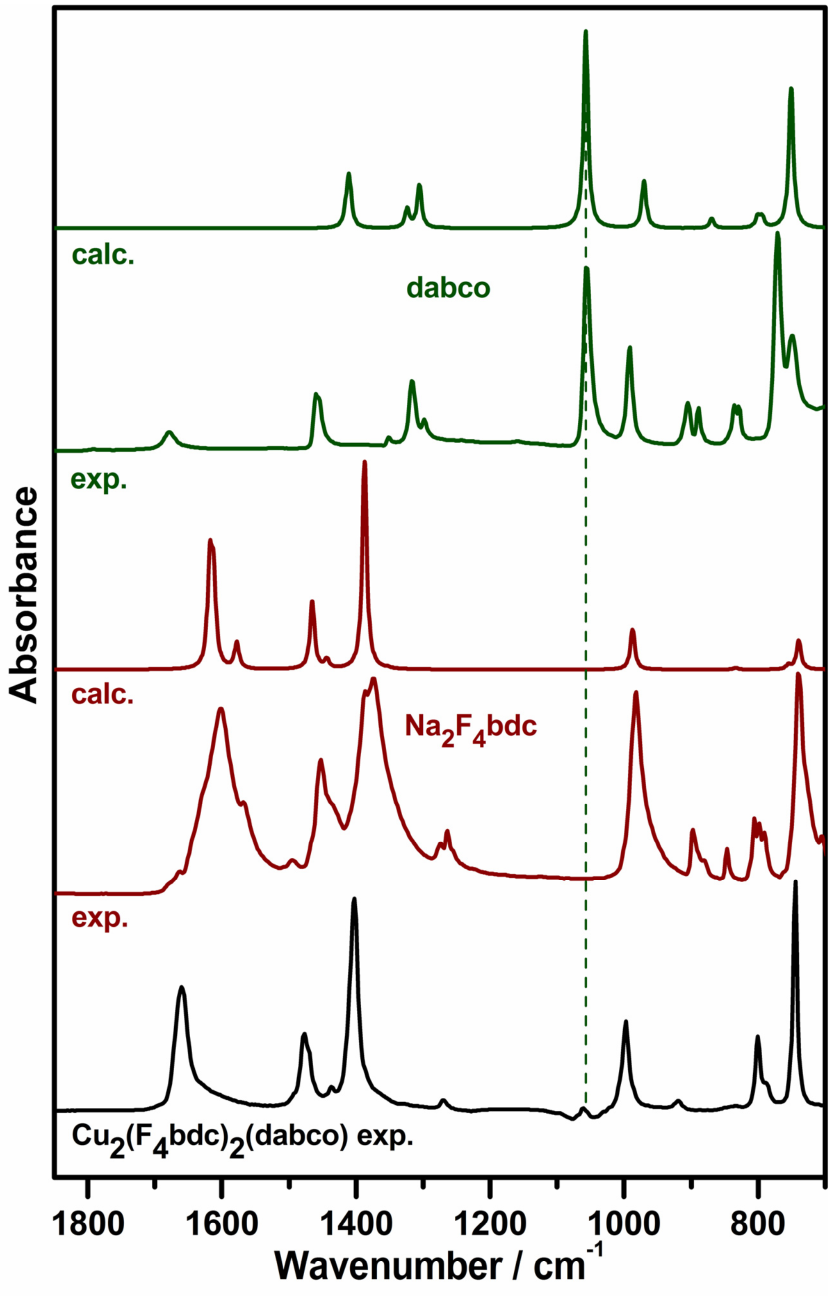

Figure S2. Infrared spectra of the $Cu_2(F_4bdc)_2$(dabco) MOF and the pillar (dabco) and linker (Na_2F_4bdc) molecules the MOF consists of. In addition to spectra recorded with an ATR unit ("exp."), for pillar and linker also DFT calculated spectra ("calc.") are given. The dashed vertical line marks the only band in die MOF spectrum that can be attributed to the dabco pillar.

Band Assignment of Infrared Spectra

Table S1: Assignment of some bands in the spectra of dabco, Na_2F_4bdc and $Cu_2(F_4bdc)_2(dabco)$. Band positions are given in cm^{-1}.

mode*	pillar (dabco)		linker (Na_2F_4bdc)		MOF
	calc.	exp.	calc.	exp.	exp.
ν COO as	-	-	1616	1602	1660
ν CF ν CC	-	-	1465	1452	1477
ν COO s	-	-	1388	1374	1403
ν CN	1057	1056	-	-	1060
ν CF	-	-	987	982	998
δ OCO δ CCC	-	-	740	740	745

*) ν: stretch, δ: in plane bend, as: asymmetric, s: symmetric

Layer-by-Layer Experiments: QCM Curves

$c(Cu^{2+})$ =	1.0	mM	c(dabco) =	0.1	mM	$c(H_2F_4bdc)$ =	0.1	mM

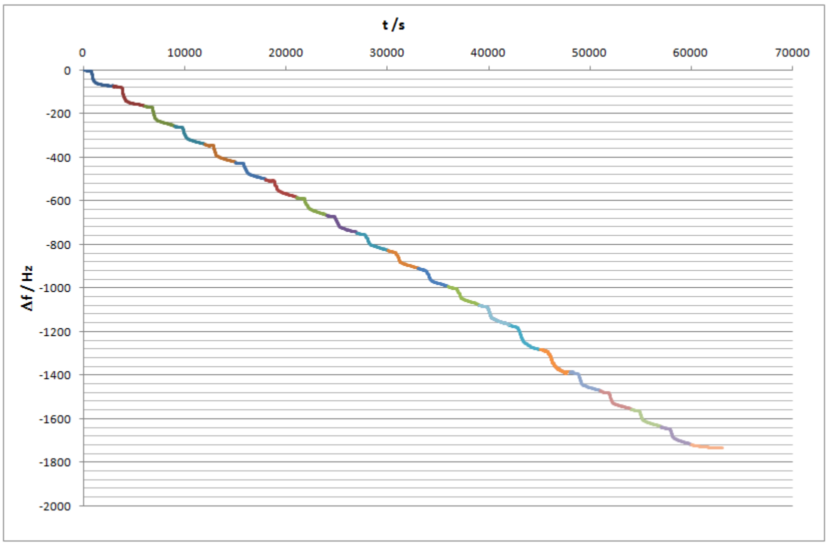

Figure S3. QCM curve of the LbL experiment with $c(Cu^{2+})$ = 1.0 mM, c(dabco) = 0.1 mM and $c(H_2F_4bdc)$ = 0.1 mM.

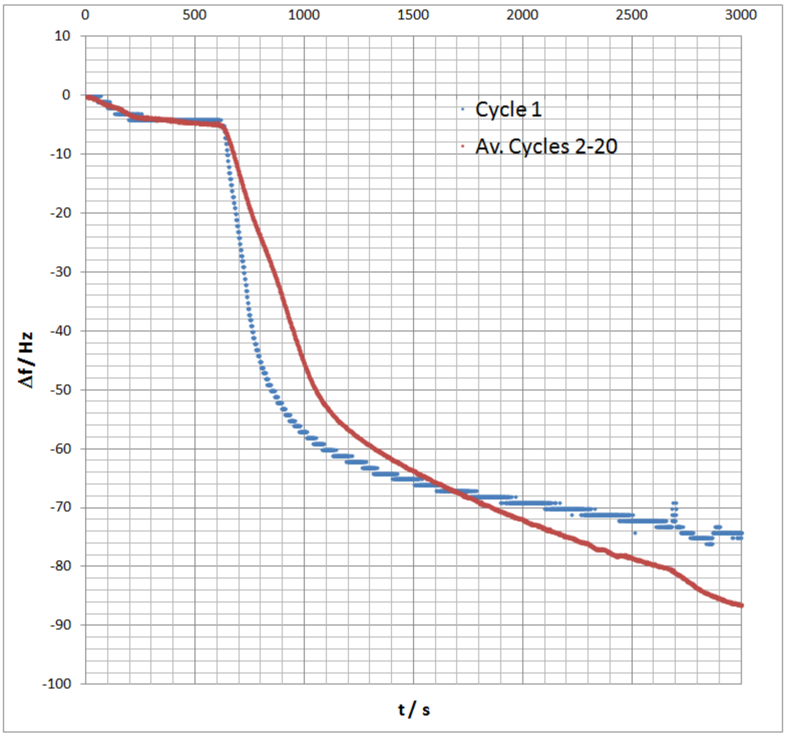

Figure S4. QCM signal (blue: cycle 1 and red: average of cycles 2-20, starting value set to $\Delta f = 0$ Hz) of the LbL experiment with $c(Cu^{2+})$ = 1.0 mM, c(dabco) = 0.1 mM and $c(H_2F_4bdc)$ = 0.1 mM.

$c(Cu^{2+})$ =	1.0	mM	c(dabco) =	0.5	mM	$c(H_2F_4bdc)$ =	0.5	mM

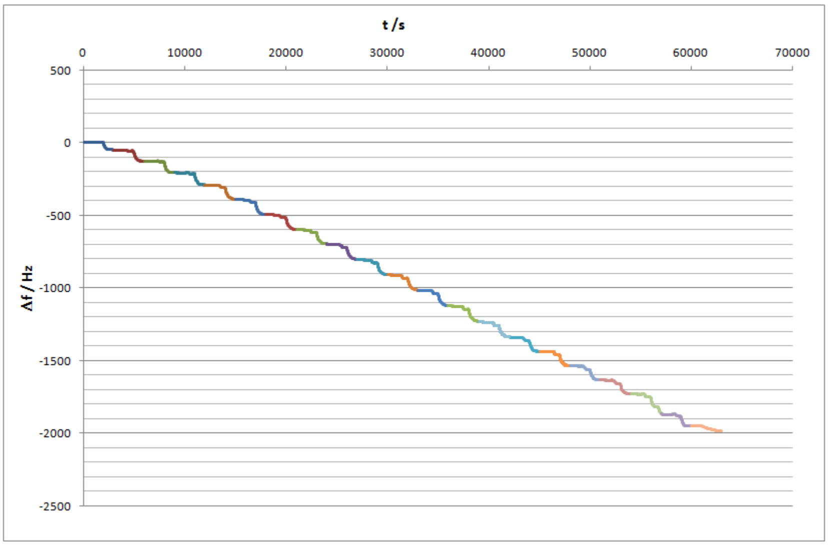

Figure S5. QCM curve of the LbL experiment with $c(Cu^{2+})$ = 1.0 mM, c(dabco) = 0.5 mM and $c(H_2F_4bdc)$ = 0.5 mM.

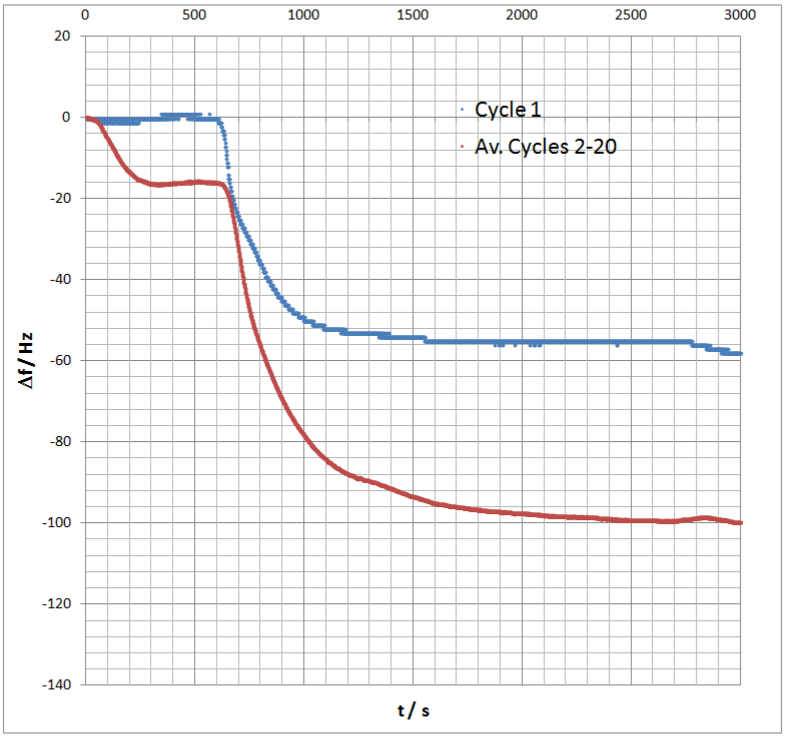

Figure S6. QCM signal (blue: cycle 1 and red: average of cycles 2-20, starting value set to Δf = 0 Hz) of the LbL experiment with $c(Cu^{2+})$ = 1.0 mM, c(dabco) = 0.5 mM and $c(H_2F_4bdc)$ = 0.5 mM.

$c(Cu^{2+})$ =	1.0	mM	c(dabco) =	1.0	mM	$c(H_2F_4bdc)$ =	1.0	mM

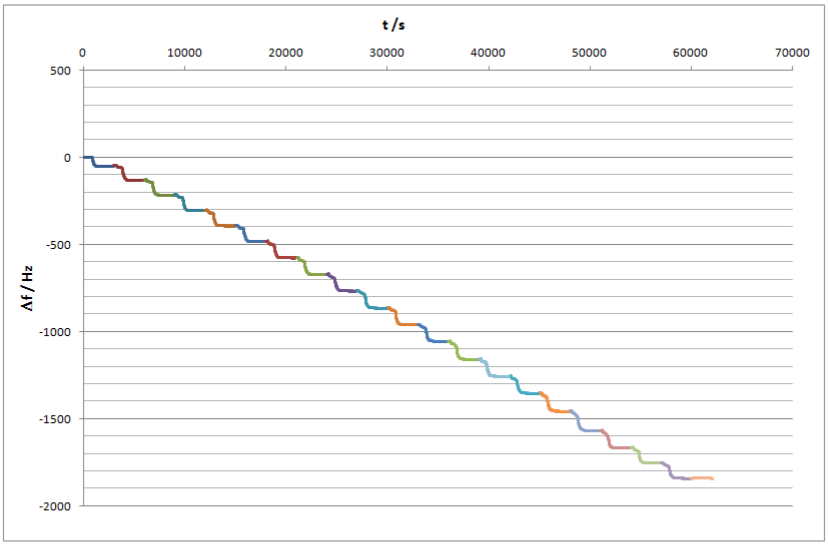

Figure S7. QCM curve of the LbL experiment with $c(Cu^{2+})$ = 1.0 mM, c(dabco) = 1.0 mM and $c(H_2F_4bdc)$ = 1.0 mM.

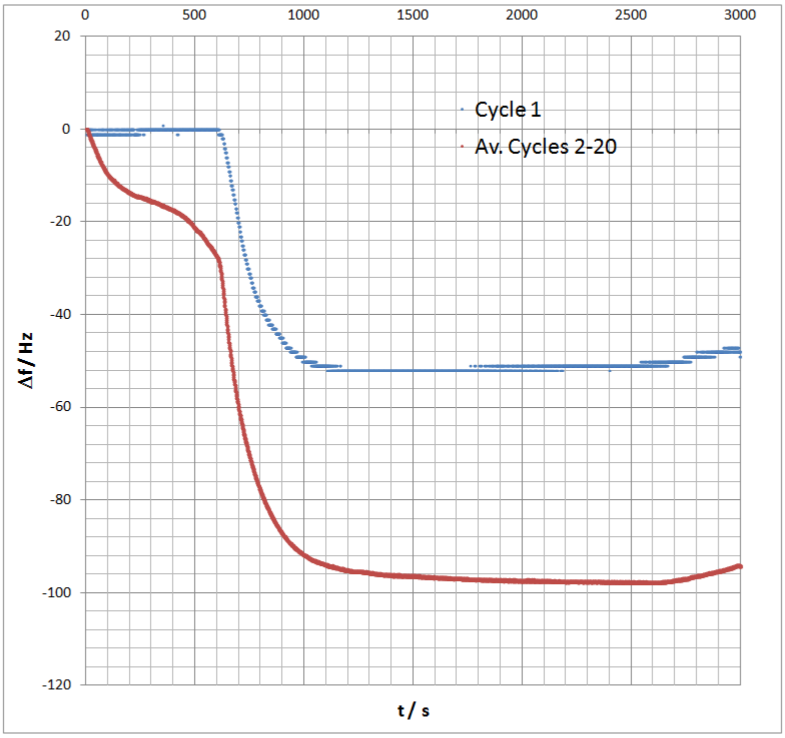

Figure S8. QCM signal (blue: cycle 1 and red: average of cycles 2-20, starting value set to Δf = 0 Hz) of the LbL experiment with $c(Cu^{2+})$ = 1.0 mM, c(dabco) = 1.0 mM and $c(H_2F_4bdc)$ = 1.0 mM.

c(Cu^{2+}) = 1.0 mM	c(dabco) = 1.5 mM	c(H_2F_4bdc) = 1.5 mM

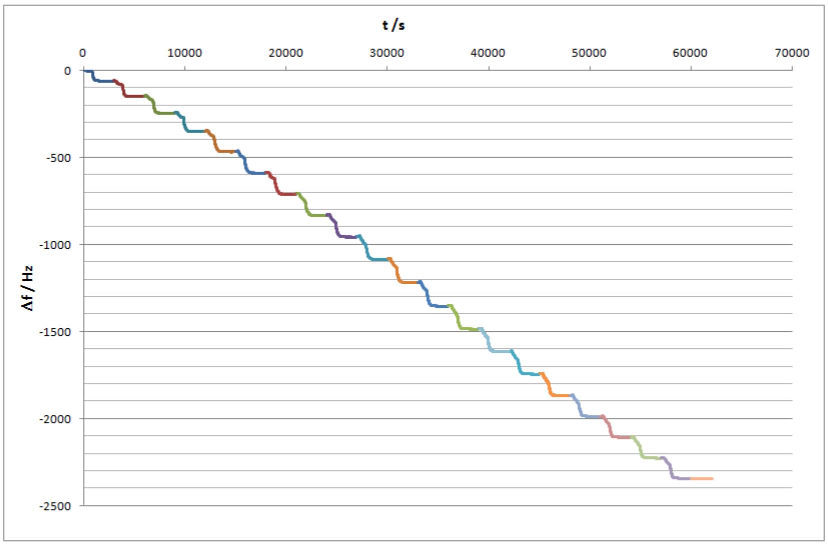

Figure S9. QCM curve of the LbL experiment with c(Cu^{2+}) = 1.0 mM, c(dabco) = 1.5 mM and c(H_2F_4bdc) = 1.5 mM.

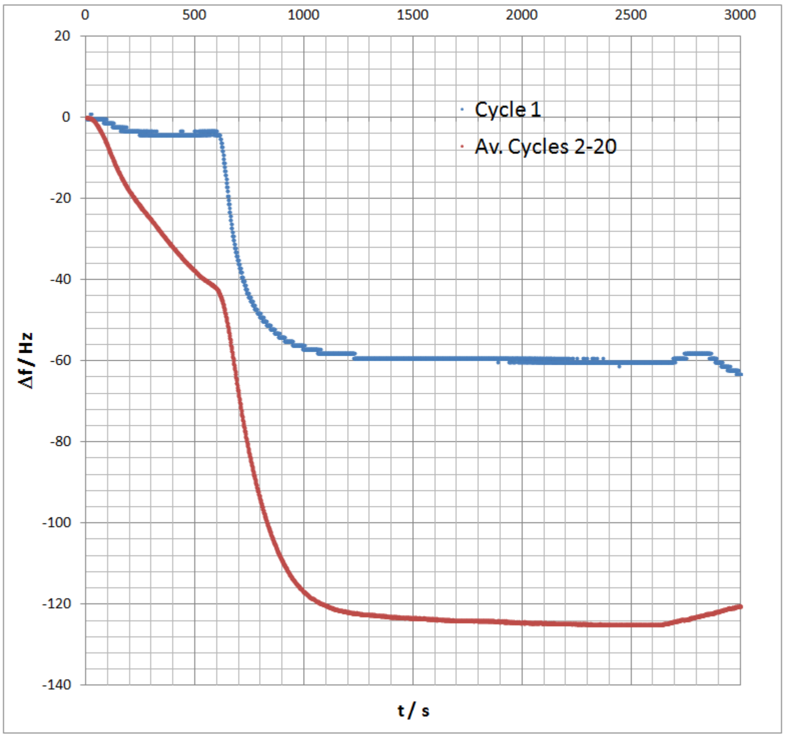

Figure S10. QCM signal (blue: cycle 1 and red: average of cycles 2-20, starting value set to Δf = 0 Hz) of the LbL experiment with c(Cu^{2+}) = 1.0 mM, c(dabco) = 1.5 mM and c(H_2F_4bdc) = 1.5 mM.

$c(Cu^{2+})$ =	1.0	mM	c(dabco) =	3.0	mM	$c(H_2F_4bdc)$ =	3.0	mM

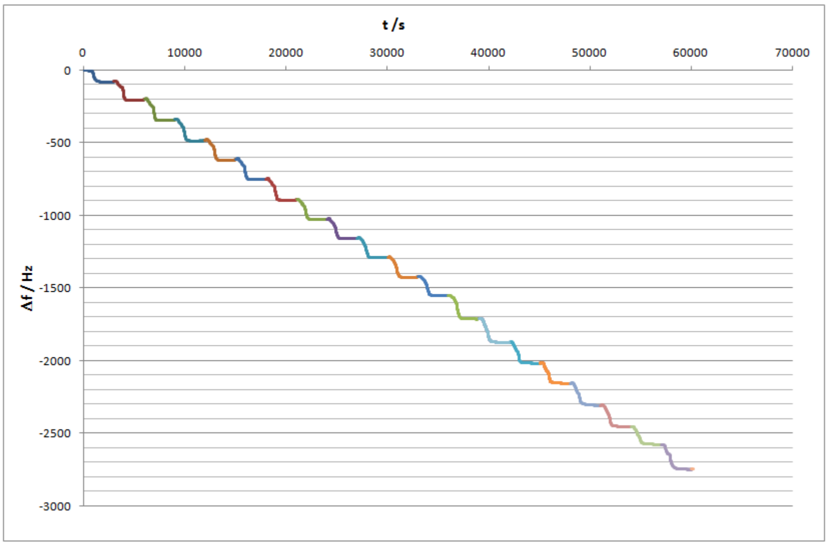

Figure S11. QCM curve of the LbL experiment with $c(Cu^{2+})$ = 1.0 mM, c(dabco) = 3.0 mM and $c(H_2F_4bdc)$ = 3.0 mM.

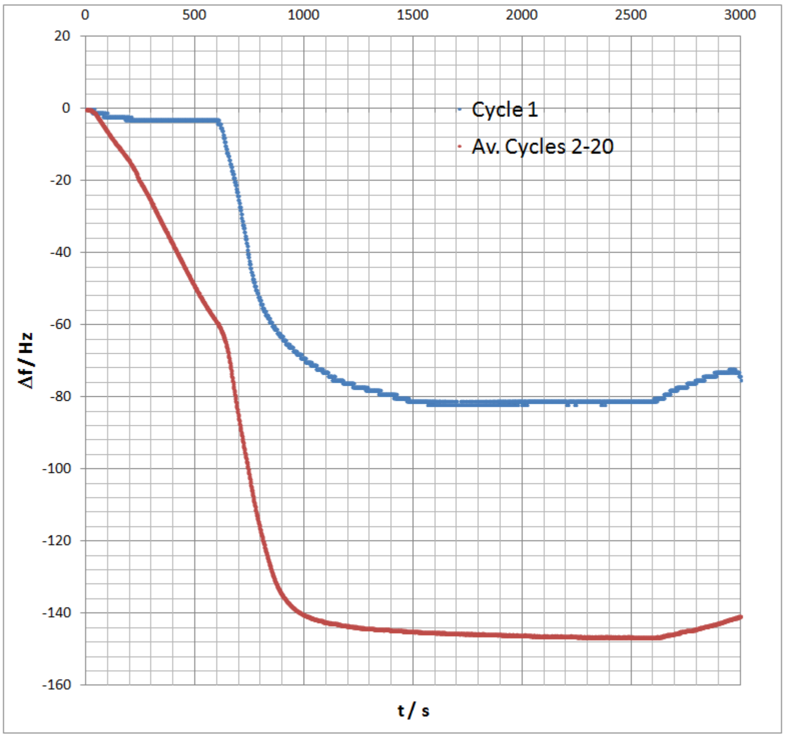

Figure S12. QCM signal (blue: cycle 1 and red: average of cycles 2-20, starting value set to Δf = 0 Hz) of the LbL experiment with $c(Cu^{2+})$ = 1.0 mM, c(dabco) = 3.0 mM and $c(H_2F_4bdc)$ = 3.0 mM.

$c(Cu^{2+})$ =	3.0	mM	c(dabco) =	0.1	mM	$c(H_2F_4bdc)$ =	0.1	mM

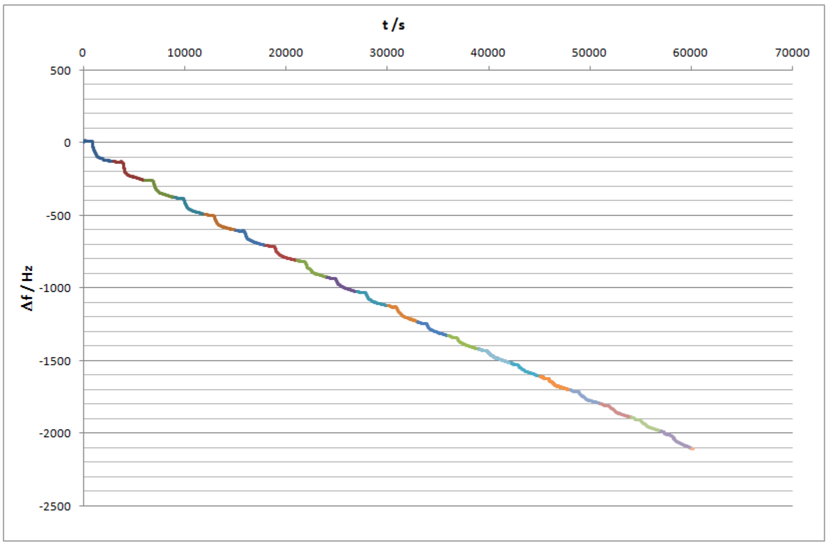

Figure S13. QCM curve of the LbL experiment with $c(Cu^{2+})$ = 3.0 mM, c(dabco) = 0.1 mM and $c(H_2F_4bdc)$ = 0.1 mM.

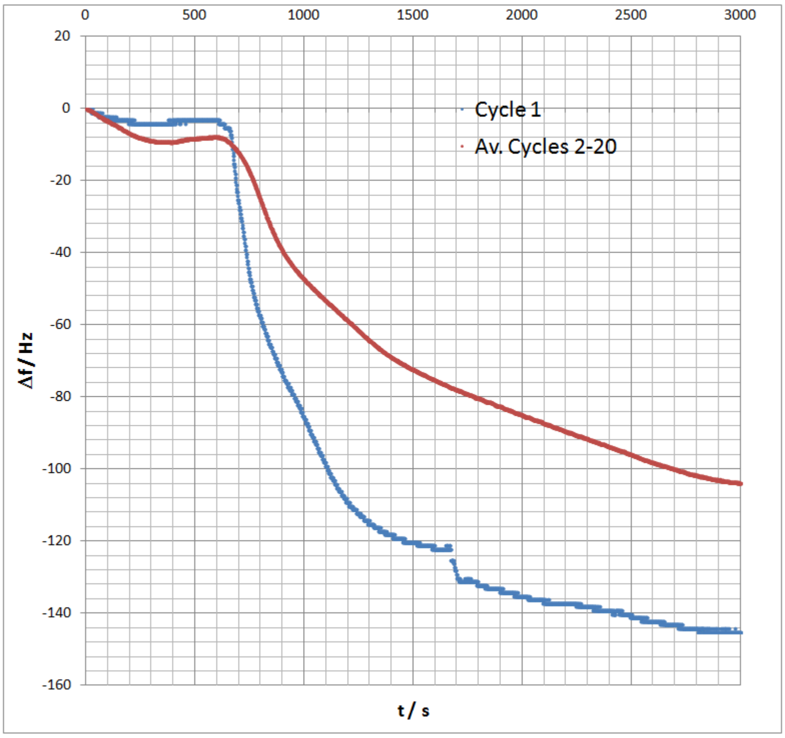

Figure S14. QCM signal (blue: cycle 1 and red: average of cycles 2-20, starting value set to Δf = 0 Hz) of the LbL experiment with $c(Cu^{2+})$ = 3.0 mM, c(dabco) = 0.1 mM and $c(H_2F_4bdc)$ = 0.1 mM.

$c(Cu^{2+})$ =	3.0	mM	c(dabco) =	3.0	mM	$c(H_2F_4bdc)$ =	3.0	mM

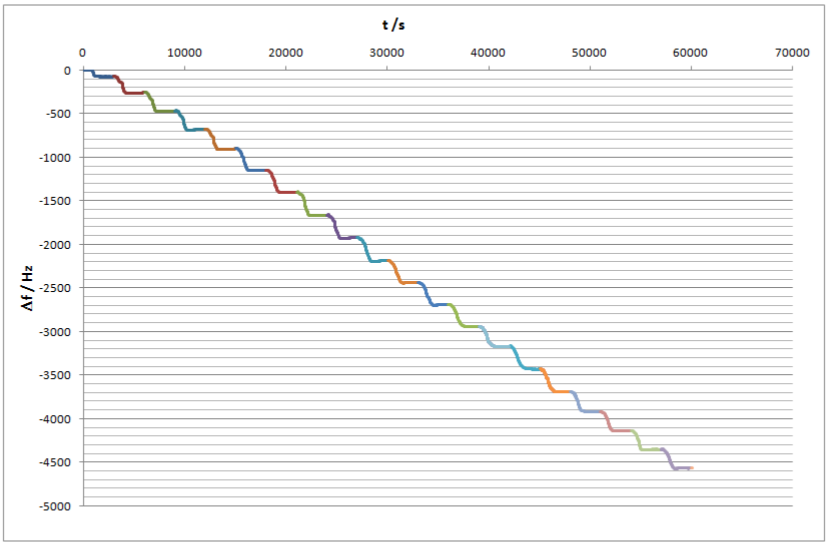

Figure S15. QCM curve of the LbL experiment with $c(Cu^{2+})$ = 3.0 mM, c(dabco) = 3.0 mM and $c(H_2F_4bdc)$ = 3.0 mM.

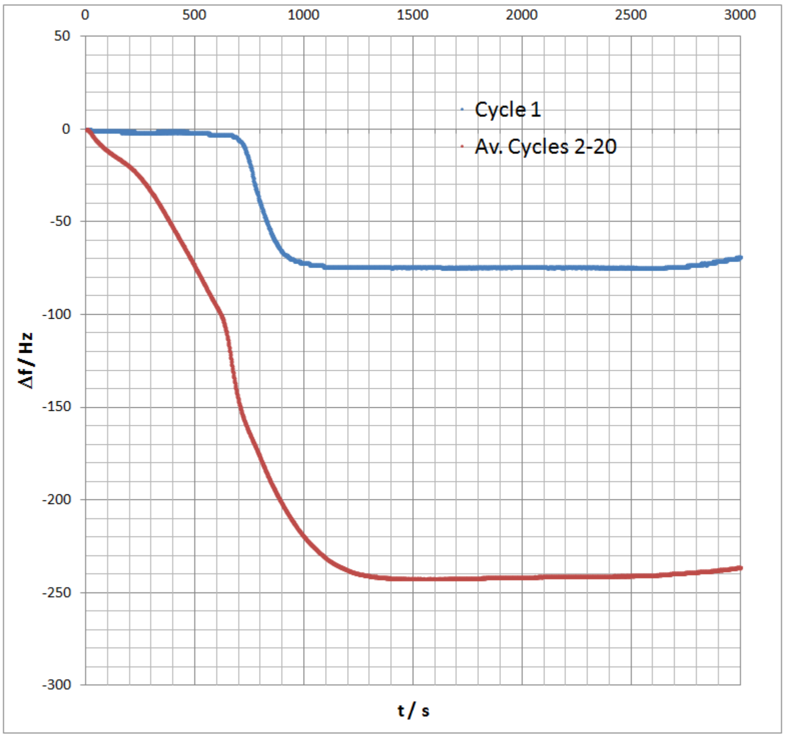

Figure S16. QCM signal (blue: cycle 1 and red: average of cycles 2-20, starting value set to Δf = 0 Hz) of the LbL experiment with $c(Cu^{2+})$ = 3.0 mM, c(dabco) = 3.0 mM and $c(H_2F_4bdc)$ = 3.0 mM.

c(Cu^{2+}) = 20.0 mM	c(dabco) = 0.1 mM	c(H_2F_4bdc) = 0.1 mM

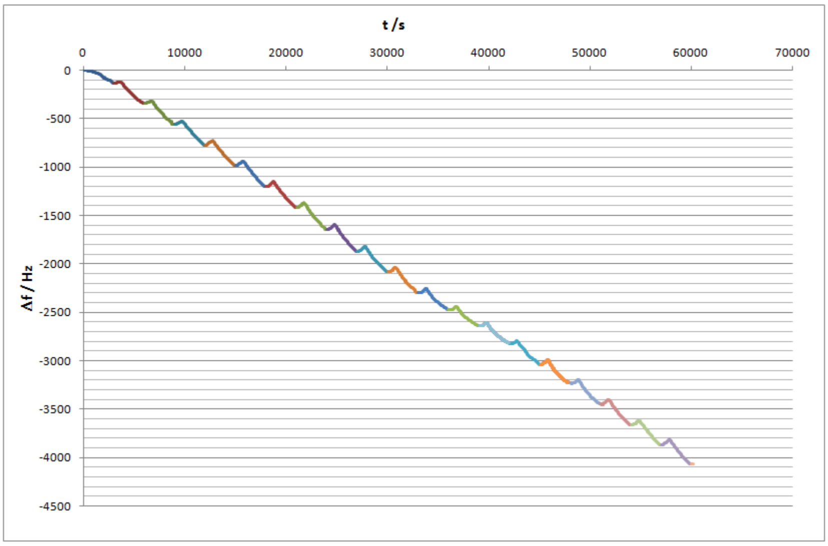

Figure S17. QCM curve of the LbL experiment with c(Cu^{2+}) = 20.0 mM, c(dabco) = 0.1 mM and c(H_2F_4bdc) = 0.1 mM.

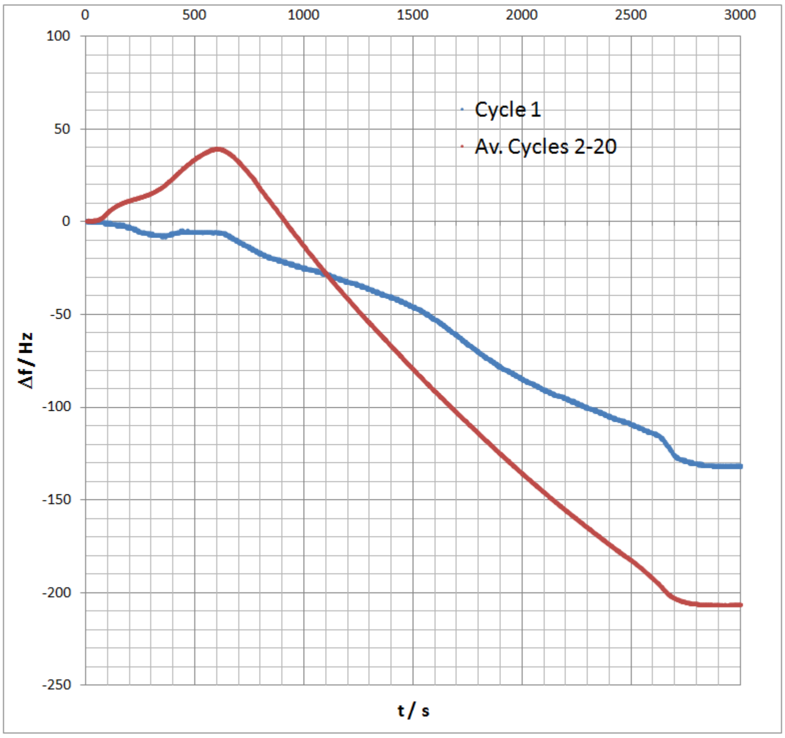

Figure S18. QCM signal (blue: cycle 1 and red: average of cycles 2-20, starting value set to Δf = 0 Hz) of the LbL experiment with c(Cu^{2+}) = 20.0 mM, c(dabco) = 0.1 mM and c(H_2F_4bdc) = 0.1 mM.

$c(Cu^{2+})$ =	20.0	mM	c(dabco) =	3.0	mM	$c(H_2F_4bdc)$ =	3.0	mM

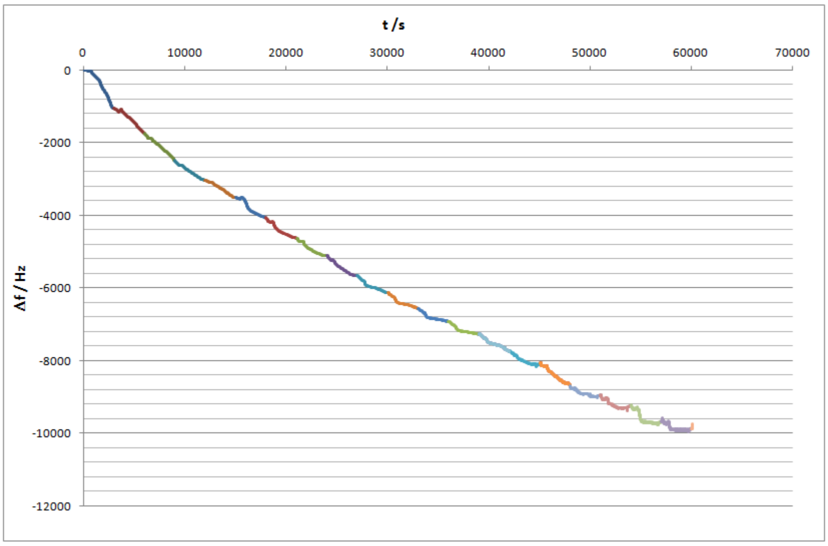

Figure S19. QCM curve of the LbL experiment with $c(Cu^{2+})$ = 20.0 mM, c(dabco) = 3.0 mM and $c(H_2F_4bdc)$ = 3.0 mM.

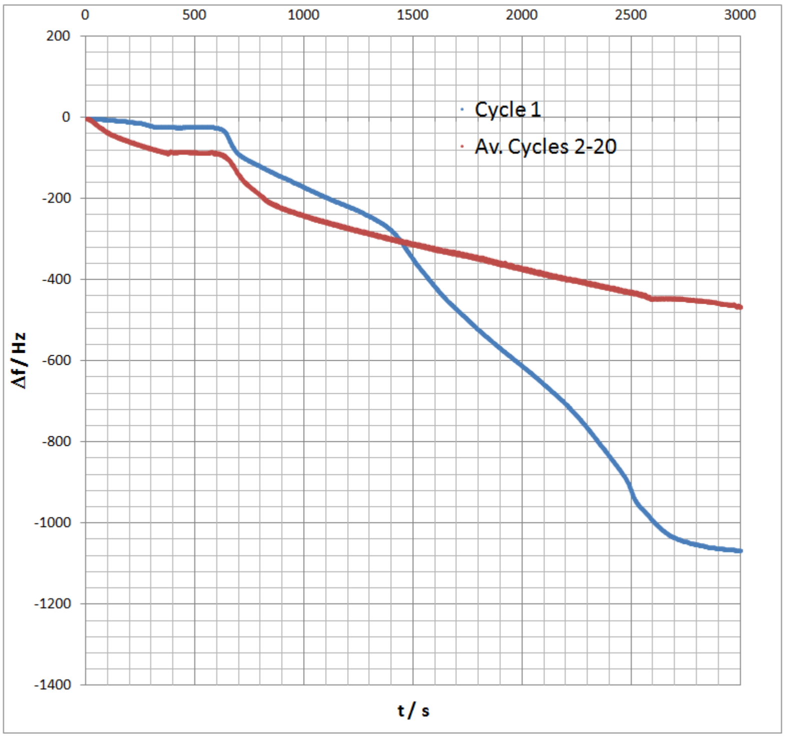

Figure S20. QCM signal (blue: cycle 1 and red: average of cycles 2-20, starting value set to Δf = 0 Hz) of the LbL experiment with $c(Cu^{2+})$ = 20.0 mM, c(dabco) = 3.0 mM and $c(H_2F_4bdc)$ = 3.0 mM.

First cycle:								
$c(Cu^{2+})$ =	1.0	mM	c(dabco) =	3.0	mM	$c(H_2F_4bdc)$ =	3.0	mM
Cycles 2-20:								
$c(Cu^{2+})$ =	1.0	mM	c(dabco) =	0.1	mM	$c(H_2F_4bdc)$ =	0.1	mM

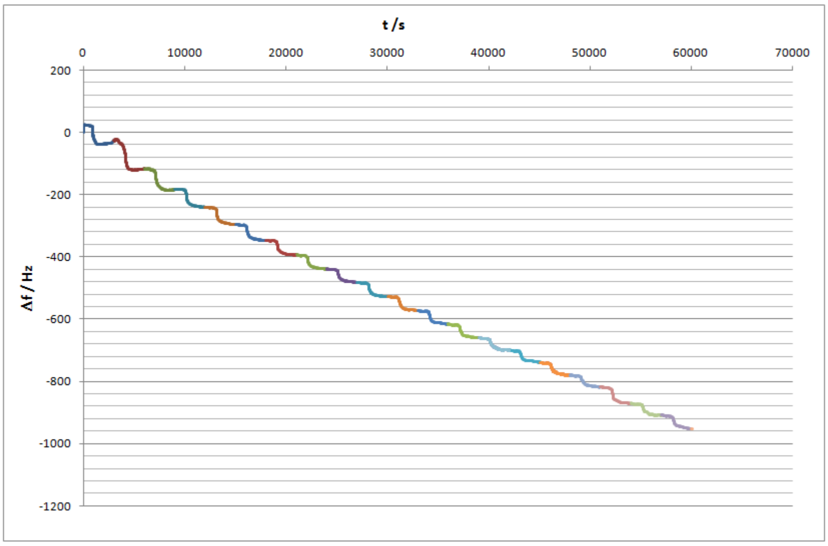

Figure S21 QCM curve of the LbL experiment with $c(Cu^{2+})$ = 1.0 mM during all cycles. In the first cycle, c(dabco) and $c(H_2F_4bdc)$ = 3.0 mM, in cycles 2-20, c(dabco) and $c(H_2F_4bdc)$ = 3.0 mM.

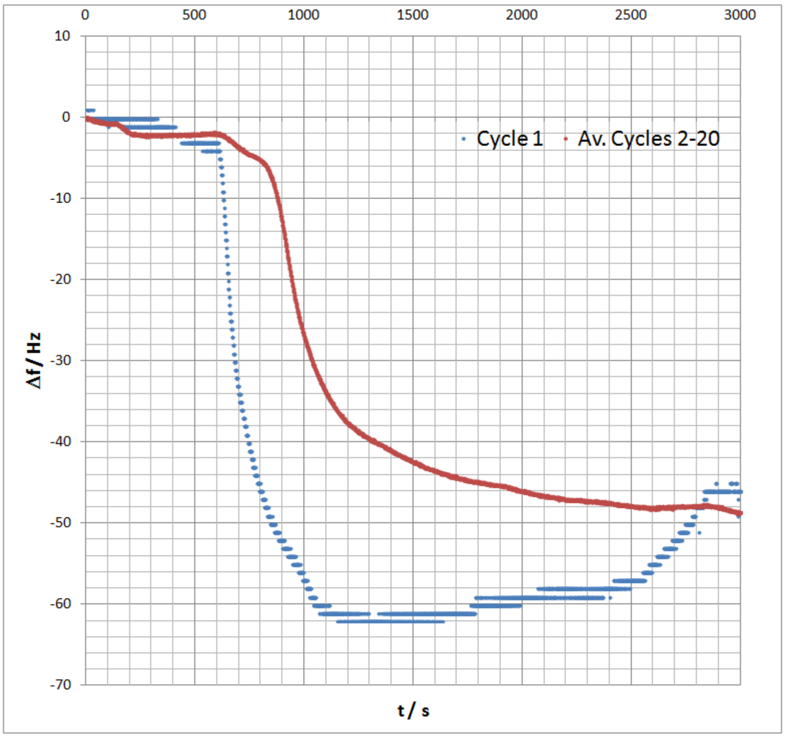

Figure S22. QCM signal (blue: cycle 1 and red: average of cycles 2-20, starting value set to Δf = 0 Hz) of the LbL experiment with $c(Cu^{2+})$ = 1.0 mM during all cycles. In the first cycle, c(dabco) and $c(H_2F_4bdc)$ = 3.0 mM, in cycles 2-20, c(dabco) and $c(H_2F_4bdc)$ = 3.0 mM.

Layer-by-Layer Experiments: SEM Images

$c(Cu^{2+})$ =	1.0	mM	c(dabco) =	0.1	mM	$c(H_2F_4bdc)$ =	0.1	mM

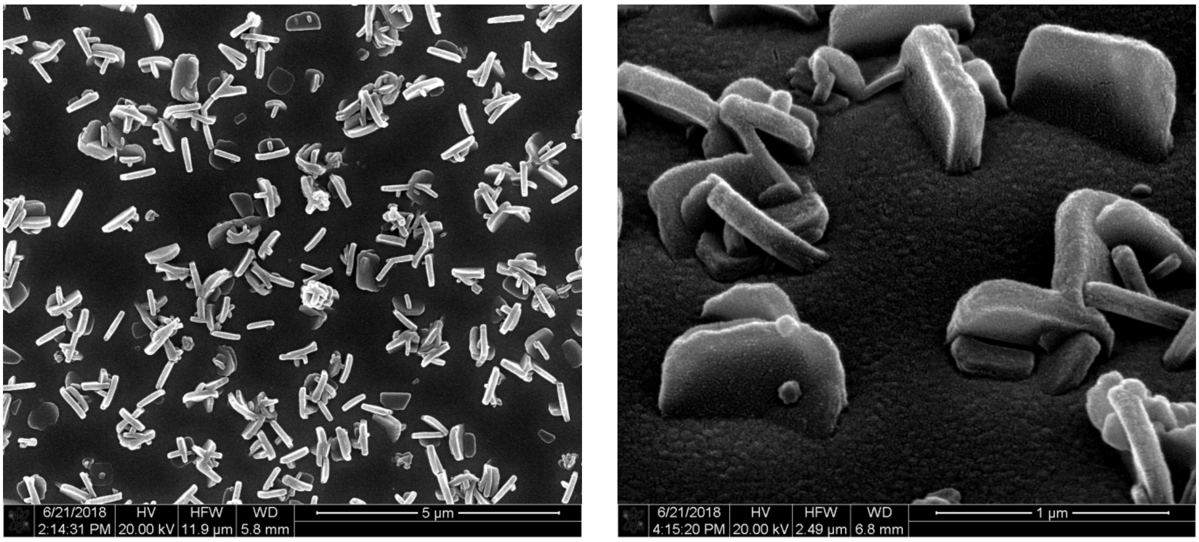

Figure S23. SEM images of the substrate after the LbL experiment with $c(Cu^{2+})$ = 1.0 mM, c(dabco) = 0.1 mM and $c(H_2F_4bdc)$ = 0.1 mM.

$c(Cu^{2+})$ =	1.0	mM	c(dabco) =	0.5	mM	$c(H_2F_4bdc)$ =	0.5	mM

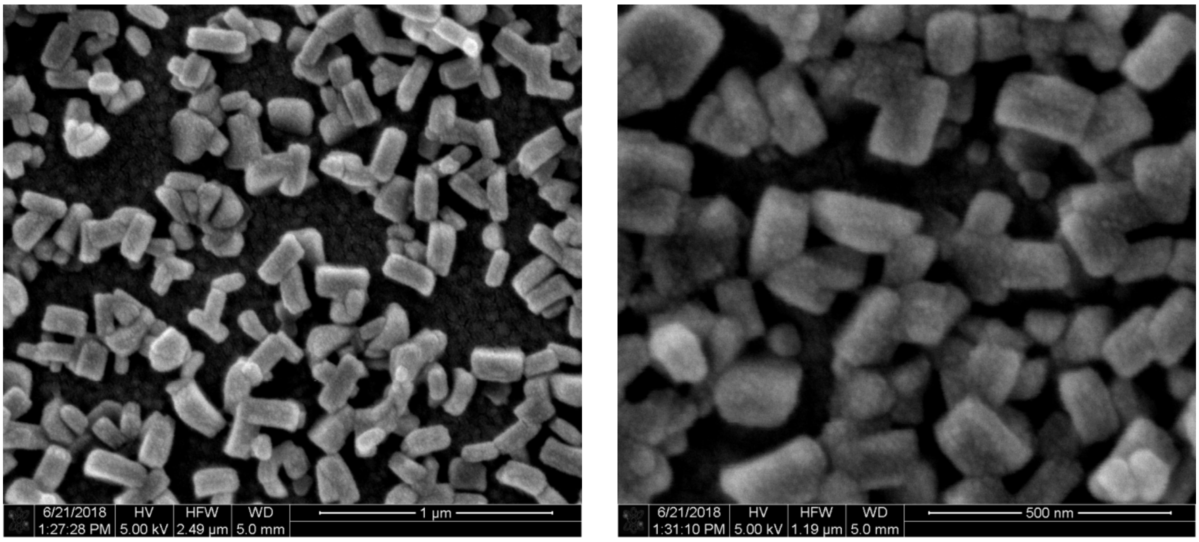

Figure S24. SEM images of the substrate after the LbL experiment with $c(Cu^{2+})$ = 1.0 mM, c(dabco) = 0.5 mM and $c(H_2F_4bdc)$ = 0.5 mM.

$c(Cu^{2+})$ =	1.0	mM	c(dabco) =	1.0	mM	$c(H_2F_4bdc)$ =	1.0	mM

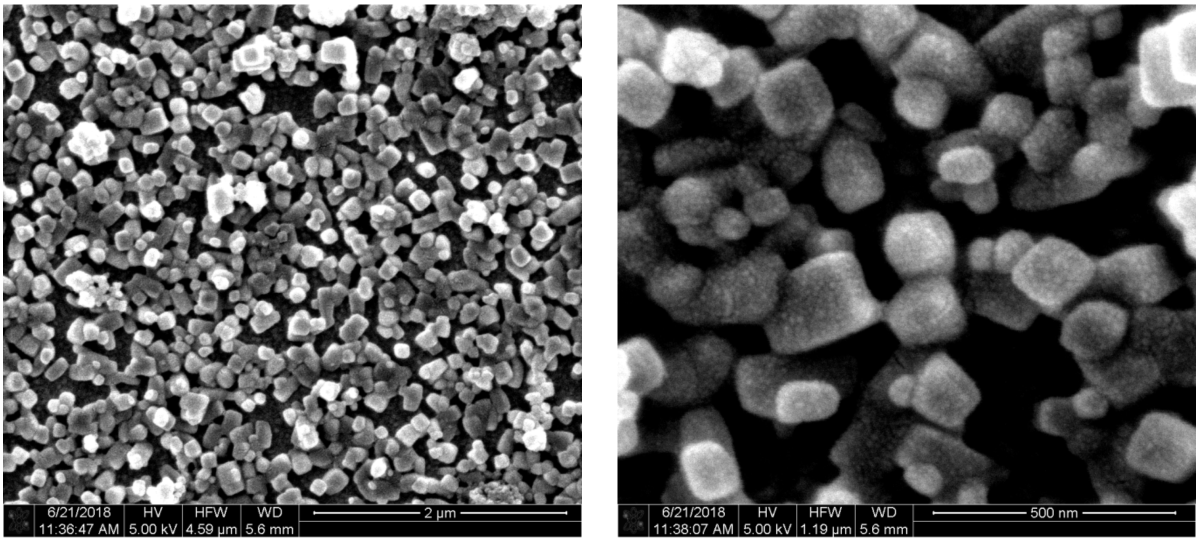

Figure S25. SEM images of the substrate after the LbL experiment with $c(Cu^{2+})$ = 1.0 mM, c(dabco) = 1.0 mM and $c(H_2F_4bdc)$ = 1.0 mM.

$c(Cu^{2+})$ =	1.0	mM	c(dabco) =	1.5	mM	$c(H_2F_4bdc)$ =	1.5	mM

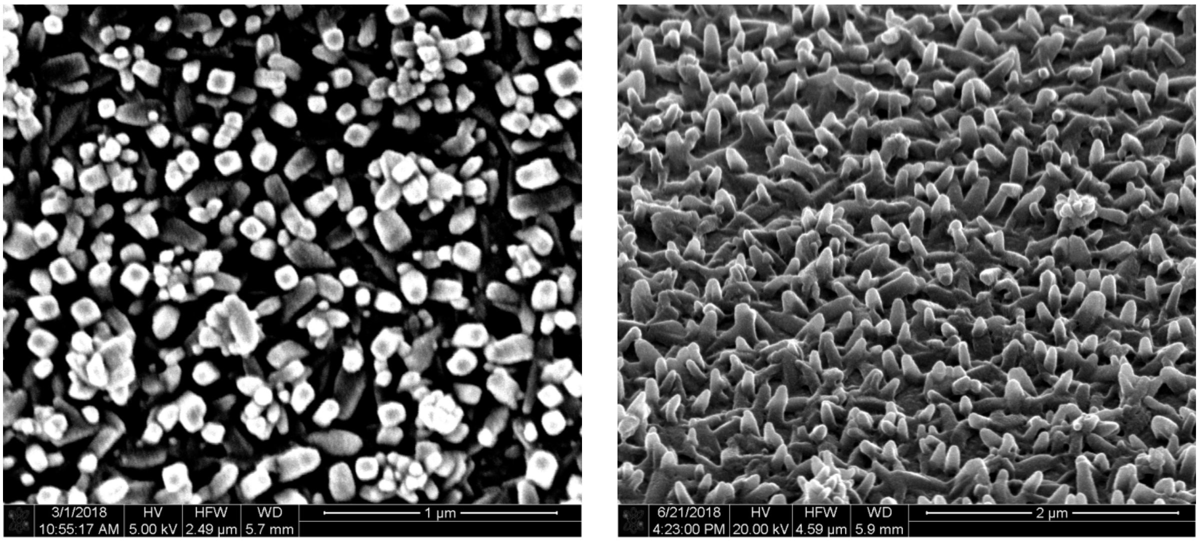

Figure S26. SEM images of the substrate after the LbL experiment with $c(Cu^{2+})$ = 1.0 mM, c(dabco) = 1.5 mM and $c(H_2F_4bdc)$ = 1.5 mM.

$c(Cu^{2+})$ = 1.0 mM	c(dabco) = 3.0 mM	$c(H_2F_4bdc)$ = 3.0 mM

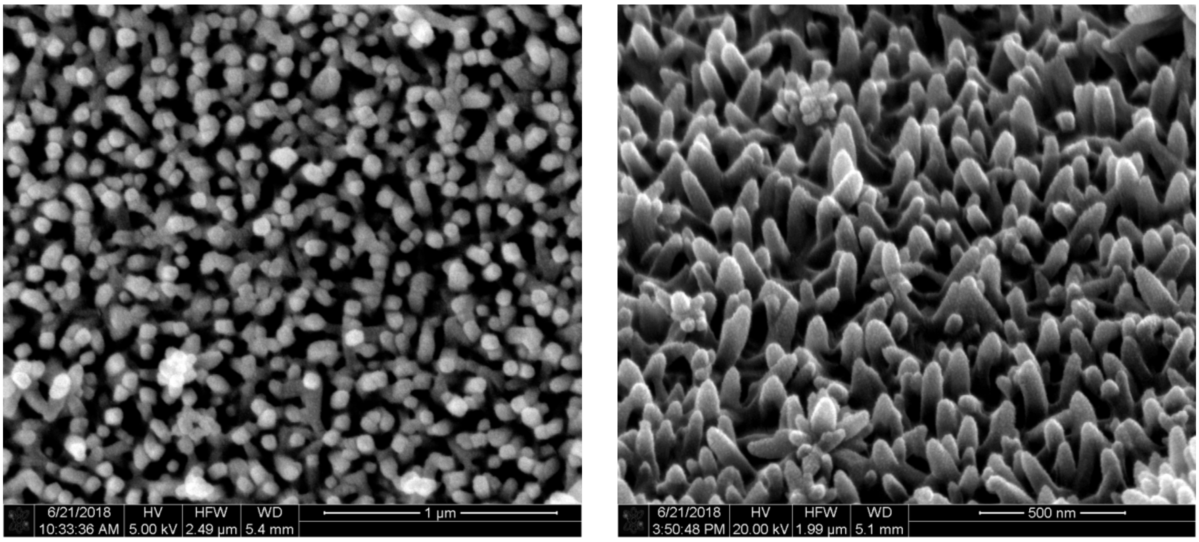

Figure S27. SEM images of the substrate after the LbL experiment with $c(Cu^{2+})$ = 1.0 mM, c(dabco) = 3.0 mM and $c(H_2F_4bdc)$ = 3.0 mM.

$c(Cu^{2+})$ = 3.0 mM	c(dabco) = 0.1 mM	$c(H_2F_4bdc)$ = 0.1 mM

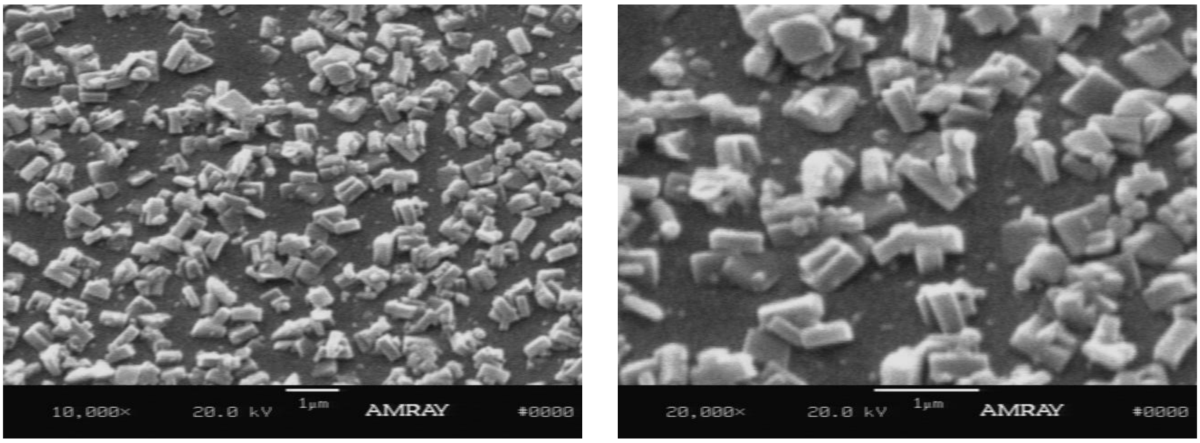

Figure S28. SEM images of the substrate after the LbL experiment with $c(Cu^{2+})$ = 3.0 mM, c(dabco) = 0.1 mM and $c(H_2F_4bdc)$ = 0.1 mM.

$c(Cu^{2+})$ = 3.0 mM	c(dabco) = 3.0 mM	$c(H_2F_4bdc)$ = 3.0 mM

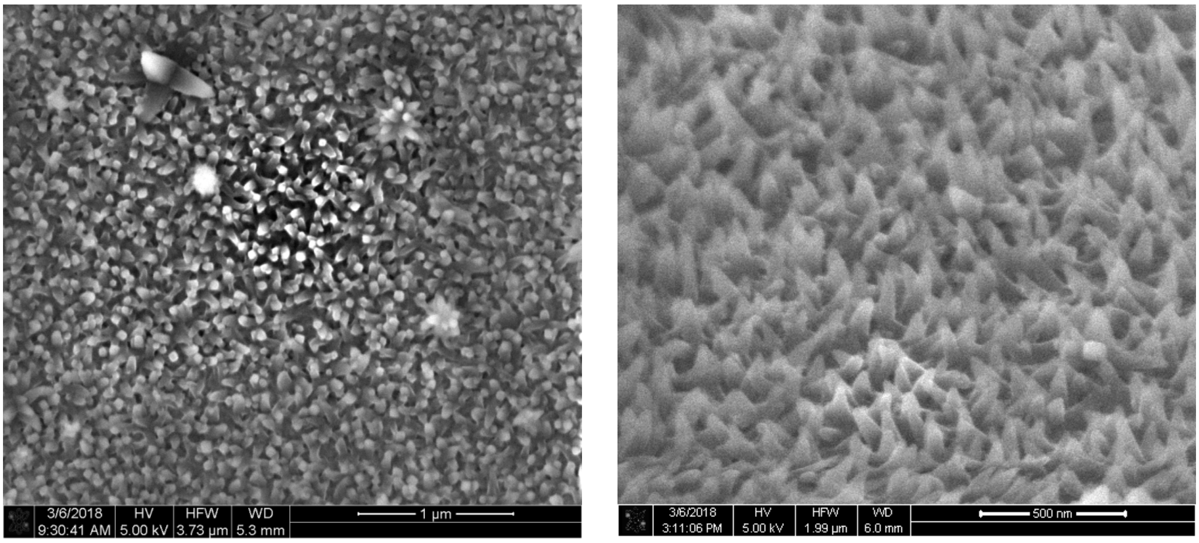

Figure S29. SEM images of the substrate after the LbL experiment with $c(Cu^{2+})$ = 3.0 mM, c(dabco) = 3.0 mM and $c(H_2F_4bdc)$ = 3.0 mM.

$c(Cu^{2+})$ = 20.0 mM	c(dabco) = 0.1 mM	$c(H_2F_4bdc)$ = 0.1 mM

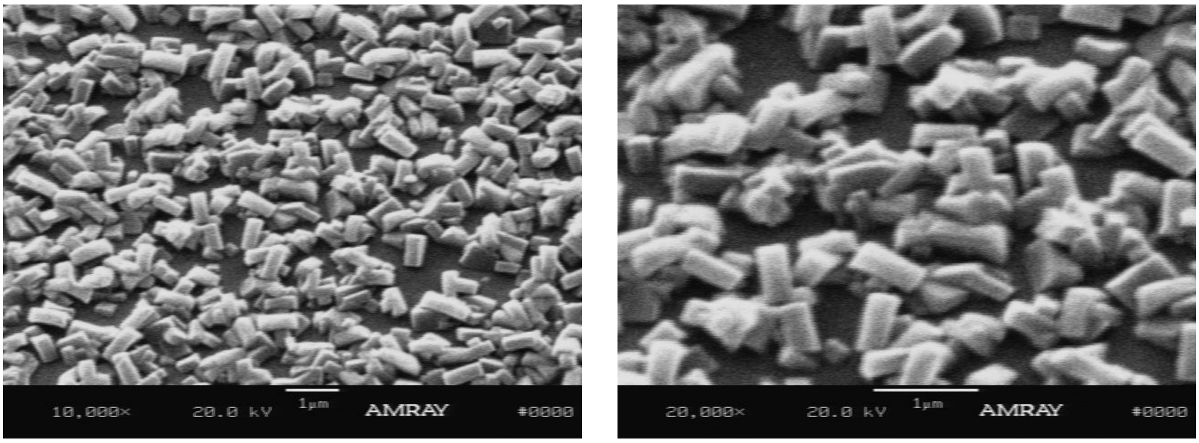

Figure S30. SEM images of the substrate after the LbL experiment with $c(Cu^{2+})$ = 20.0 mM, c(dabco) = 0.1 mM and $c(H_2F_4bdc)$ = 0.1 mM.

$c(Cu^{2+})$ =	20.0	mM	c(dabco) =	3.0	mM	$c(H_2F_4bdc)$ =	3.0	mM

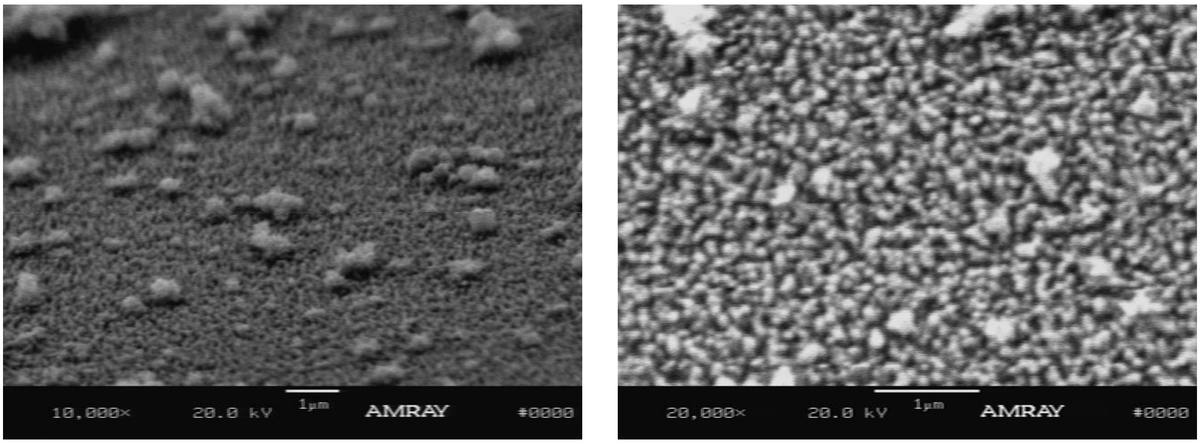

Figure S31. SEM images of the substrate after the LbL experiment with $c(Cu^{2+})$ = 20.0 mM, c(dabco) = 3.0 mM and $c(H_2F_4bdc)$ = 3.0 mM.

First cycle:								
$c(Cu^{2+})$ =	1.0	mM	c(dabco) =	3.0	mM	$c(H_2F_4bdc)$ =	3.0	mM
Cycles 2-20:								
$c(Cu^{2+})$ =	1.0	mM	c(dabco) =	0.1	mM	$c(H_2F_4bdc)$ =	0.1	mM

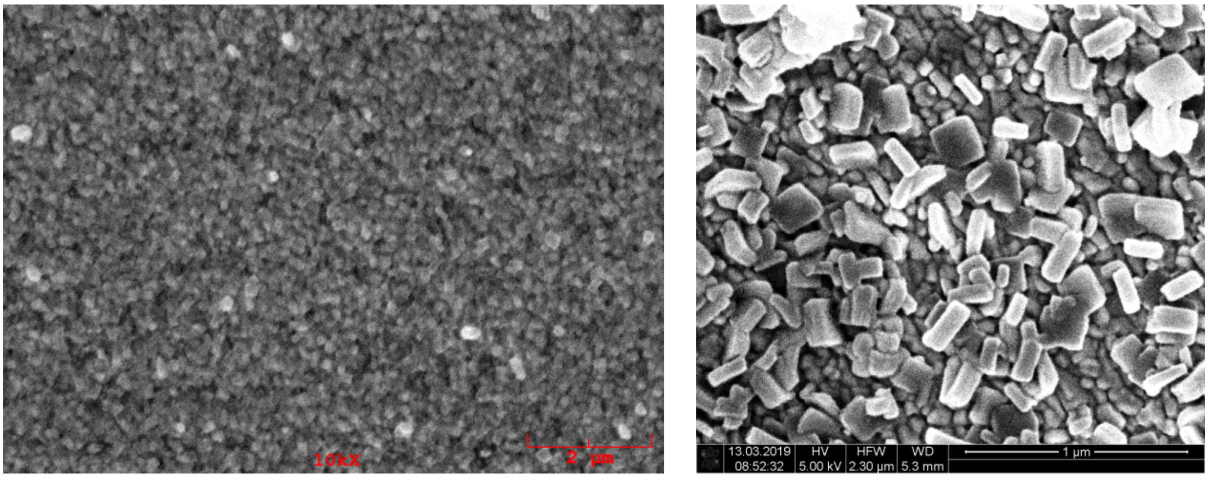

Figure S32. SEM images of the substrate after the LbL experiment with $c(Cu^{2+})$ = 1.0 mM during all cycles. In the first cycle, c(dabco) and $c(H_2F_4bdc)$ = 3.0 mM, in cycles 2-20, c(dabco) and $c(H_2F_4bdc)$ = 0.1 mM.

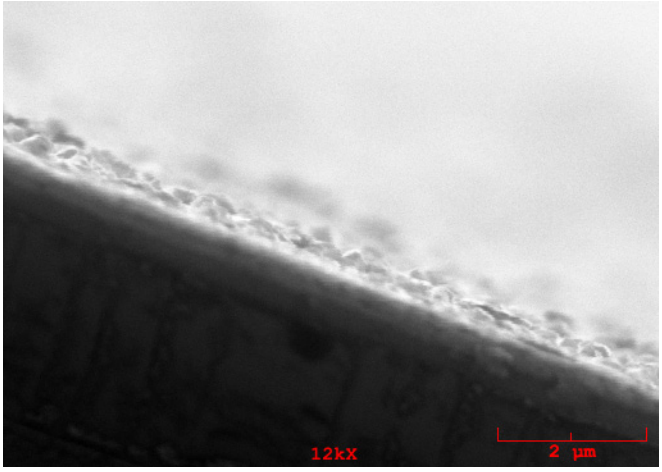

Figure S32a. SEM image (side view) of the substrate after the LbL experiment with $c(Cu^{2+})$ = 1.0 mM during all cycles. In the first cycle, c(dabco) and c(H2F4bdc) = 3.0 mM, in cycles 2-20, c(dabco) and $c(H_2F_4bdc)$ = 0.1 mM. A rough estimation of the layer thickness results in ca. 250 nm.

X-ray Diffraction Data of $Cu_2(F_4bdc)_2(dabco)$ Powder and SURMOFs

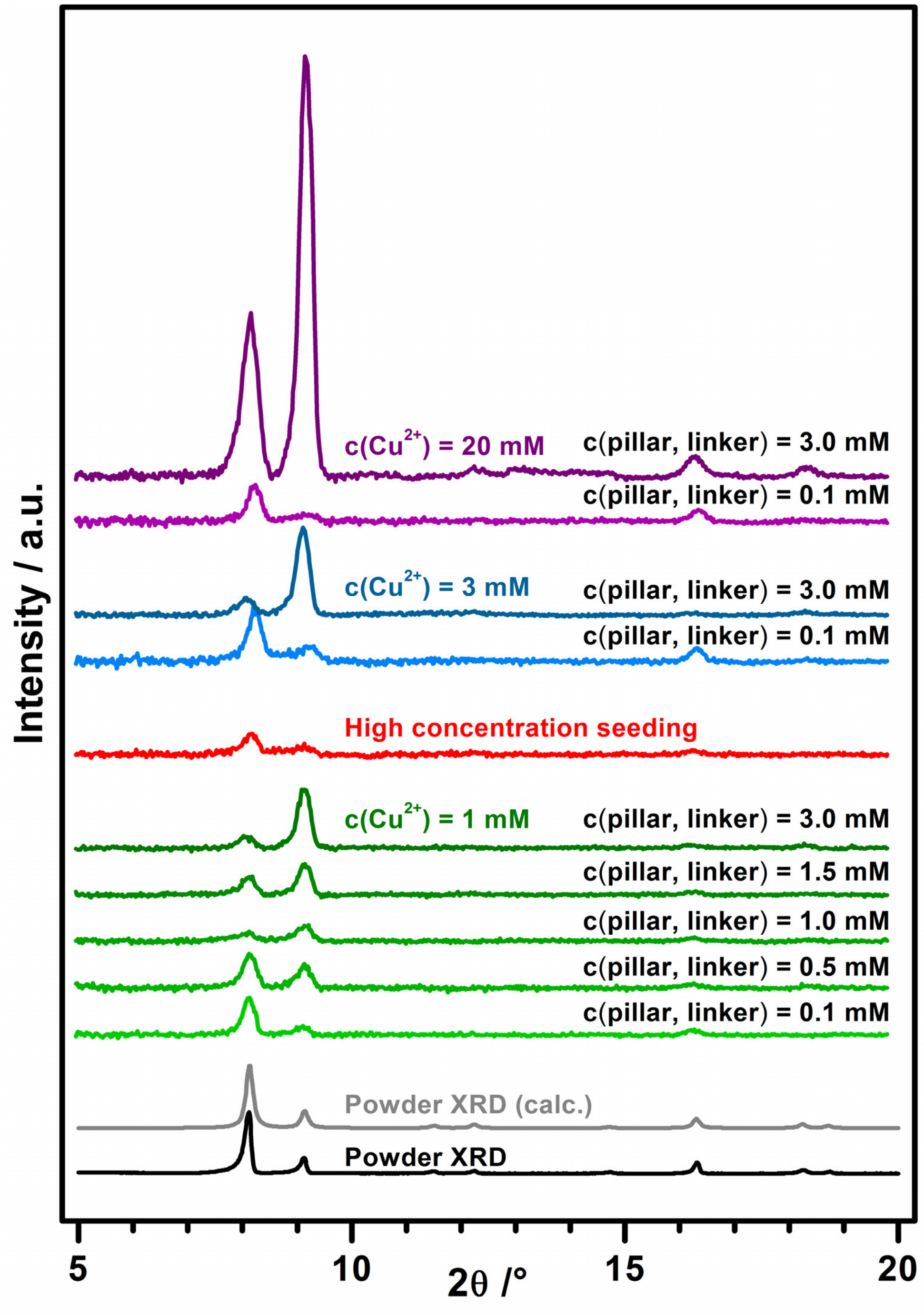

Figure S33. Experimental XRD patterns of the $Cu_2(F_4bdc)_2(dabco)$ SURMOFs on PPP1@Au and bulk MOF as well as a powder XRD pattern of $Cu_2(F_4bdc)_2(dabco)$ calculated from crystal structure data.[1] All SURMOF XRD data are displayed at the same scale of arbitrary intensity units, the powder XRD intensities are scaled such that they appear of similar intensity. Data are shifted in height. The SURMOF data are baseline-corrected and their 2θ values are scaled by a factor of 0.99 to compensate adjustment errors in the diffractometer. "High concentration seeding": 1st cycle with $c_{Cu^{2+}}$ = 1 mM, c(pillar, linker) = 3.0 mM and cycles 2-20: $c_{Cu^{2+}}$ = 1 mM, c(pillar, linker) = 0.1 mM.

Infrared Spectra of $Cu_2(F_4bdc)_2$(dabco) Bulk and SURMOF Samples

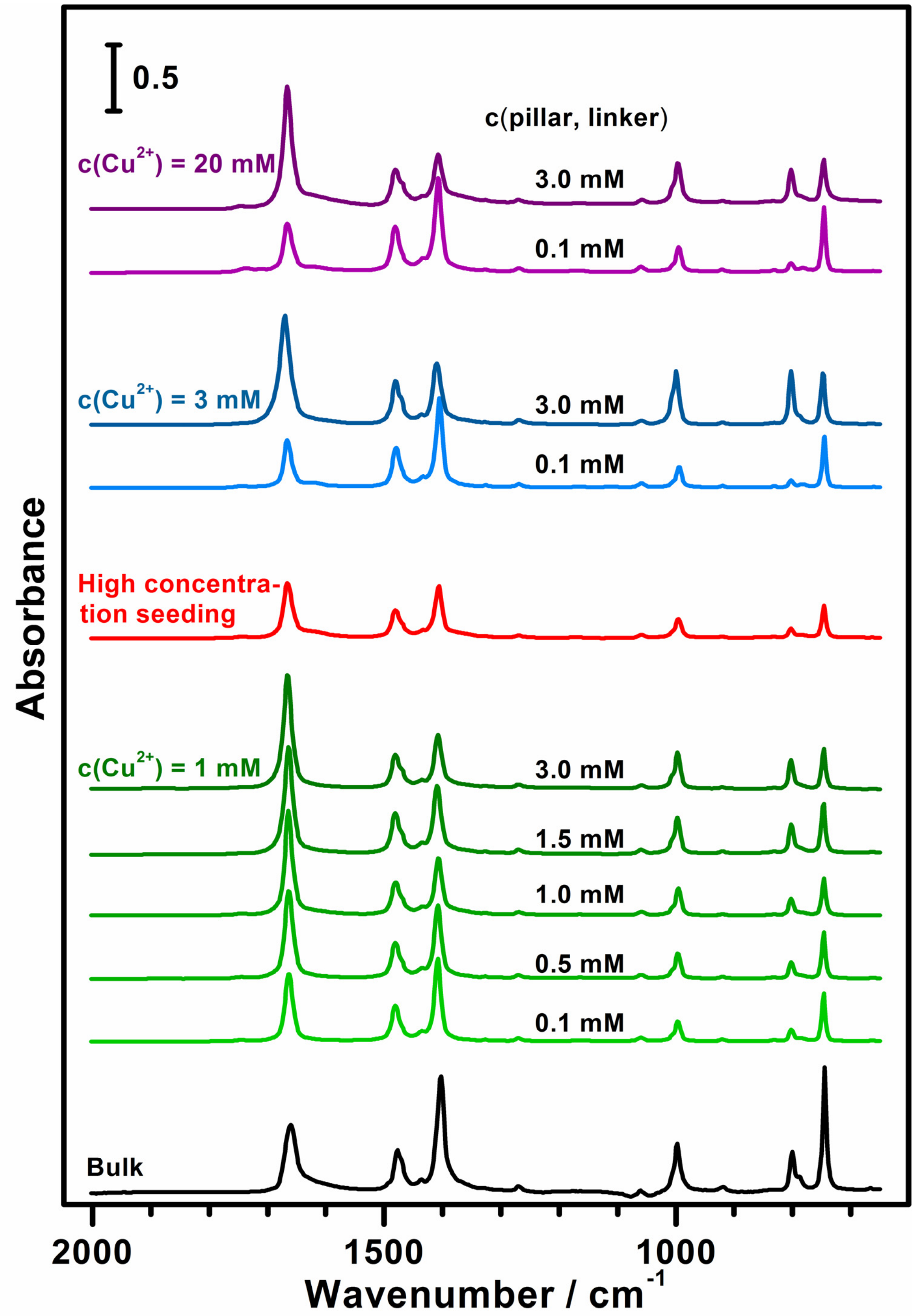

Figure S34. Infrared Spectra of the $Cu_2(F_4bdc)_2$(dabco) SURMOFs on PPP1@Au and bulk MOF. All SURMOF IR data are displayed at the same scale of absorption unit (see given scale bar), the absorbance signal of the bulk MOF spectrum is shown in arbitrary units. Data are shifted in height.

QCM: LbL Relative Mass Changes

Mass Changes after 20 LbL Cycles

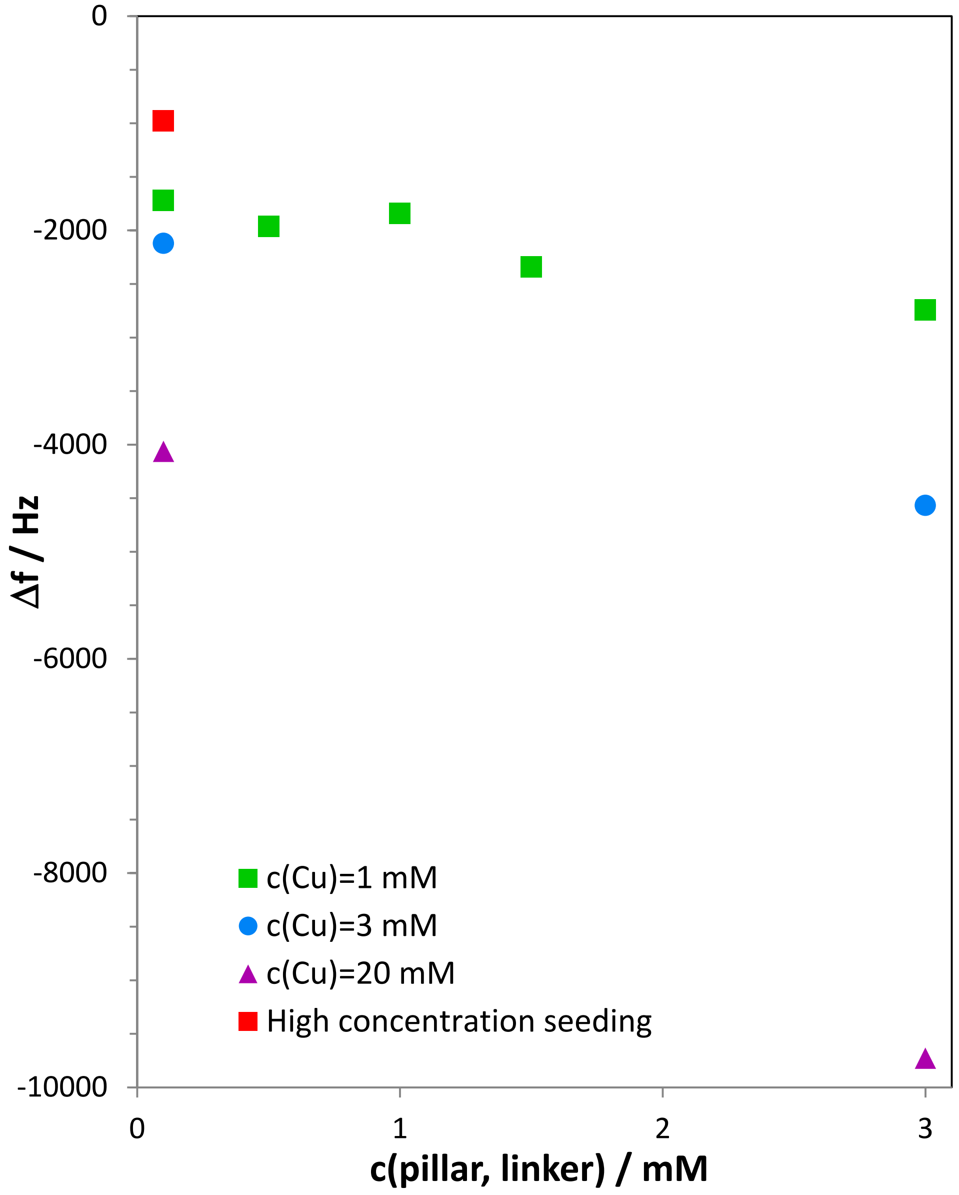

Figure S35. Relative mass changes, given by frequency changes Δf, after 20 layer-by-layer steps, in dependence on the concentration of the of pillar/linker solution. "High concentration seeding": 1st cycle with $c_{Cu^{2+}}$ = 1 mM, c(pillar, linker) = 3.0 mM and cycles 2-20: $c_{Cu^{2+}}$ = 1 mM, c(pillar, linker) = 0.1 mM.

Full Cycle Mass Changes

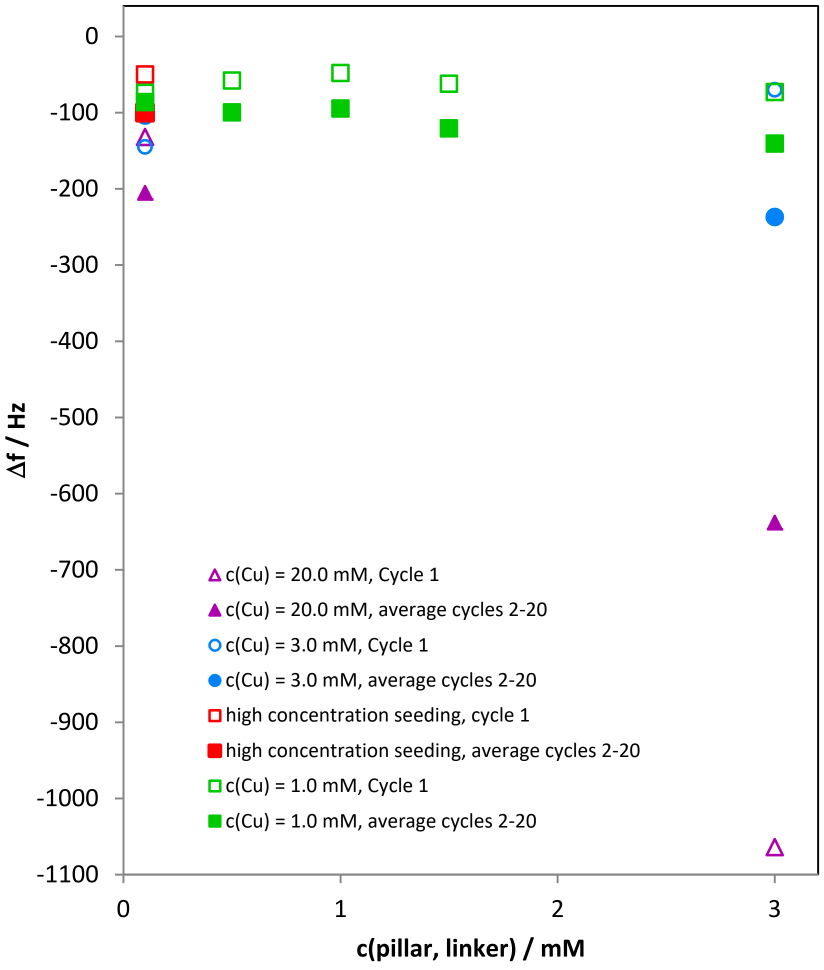

Figure S36. Relative mass changes, given by frequency changes Δf, during single layer-by-layer steps (i.e., immersion into Cu^{2+} and pillar/linker solutions and respective rinsing with solvent), in dependence on the concentration of the of pillar/linker solution. Full symbols: cycle 1, empty symbols: averages of cycles 2-20. "High concentration seeding": 1st cycle with $c_{Cu^{2+}}$ = 1 mM, c(pillar, linker) = 3.0 mM and cycles 2-20: $c_{Cu^{2+}}$ = 1 mM, c(pillar, linker) = 0.1 mM.

Mass Changes during Rinsing with Cu Solution

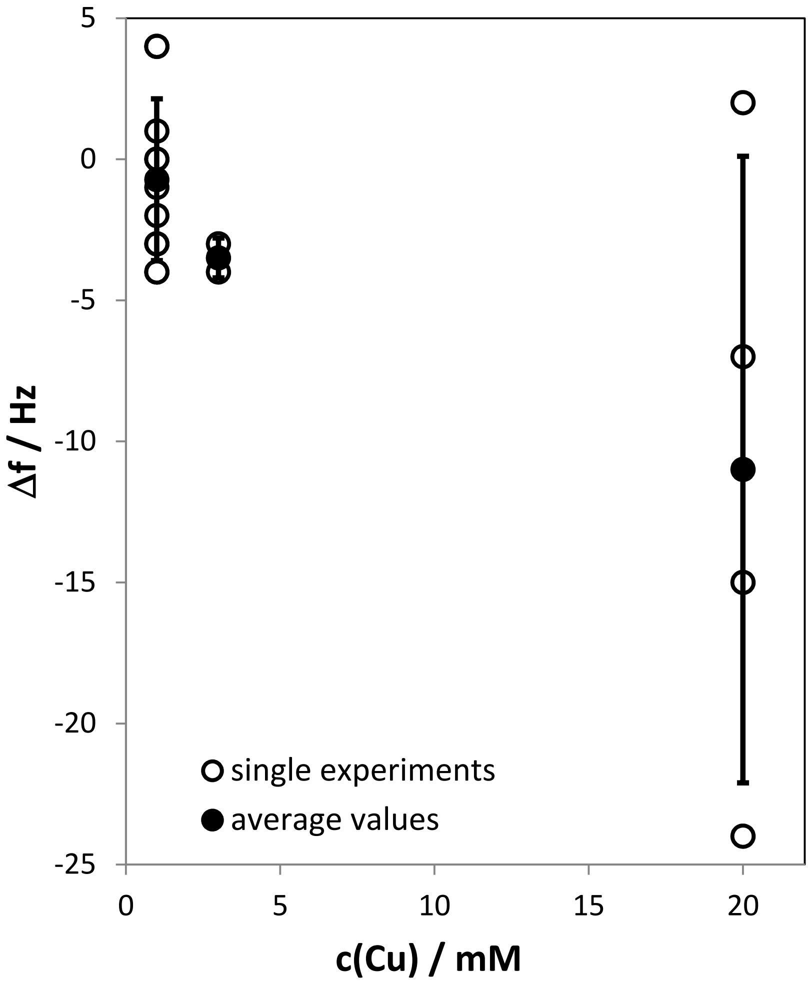

Figure S37. Relative mass changes *during the first cycle*, given by frequency changes Δf, upon rinsing with Cu^{2+} solution and subsequent washing with pure ethanol in dependence on the Cu^{2+} solution concentration, before the first contact of the samples with pillar and linker molecules.

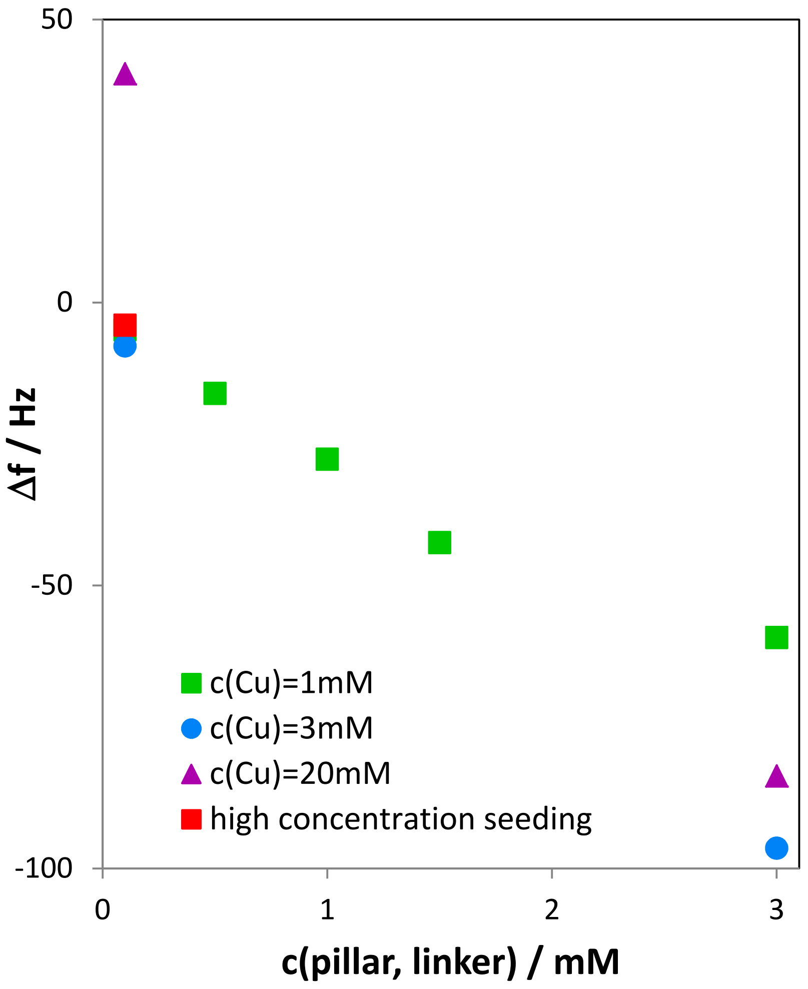

Figure S38. Relative mass changes - *average of cycles 2-20 -,* given by frequency changes Δf, upon rinsing with Cu^{2+} solution and subsequent washing with pure ethanol in dependence on the Cu^{2+} and pillar/linker solution concentrations. "High concentration seeding": 1st cycle with $c_{Cu^{2+}}$ = 1 mM, c(pillar, linker) = 3.0 mM and cycles 2-20: $c_{Cu^{2+}}$ = 1 mM, c(pillar, linker) = 0.1 mM.

Mass Changes during Rinsing with Pillar/Linker Solution

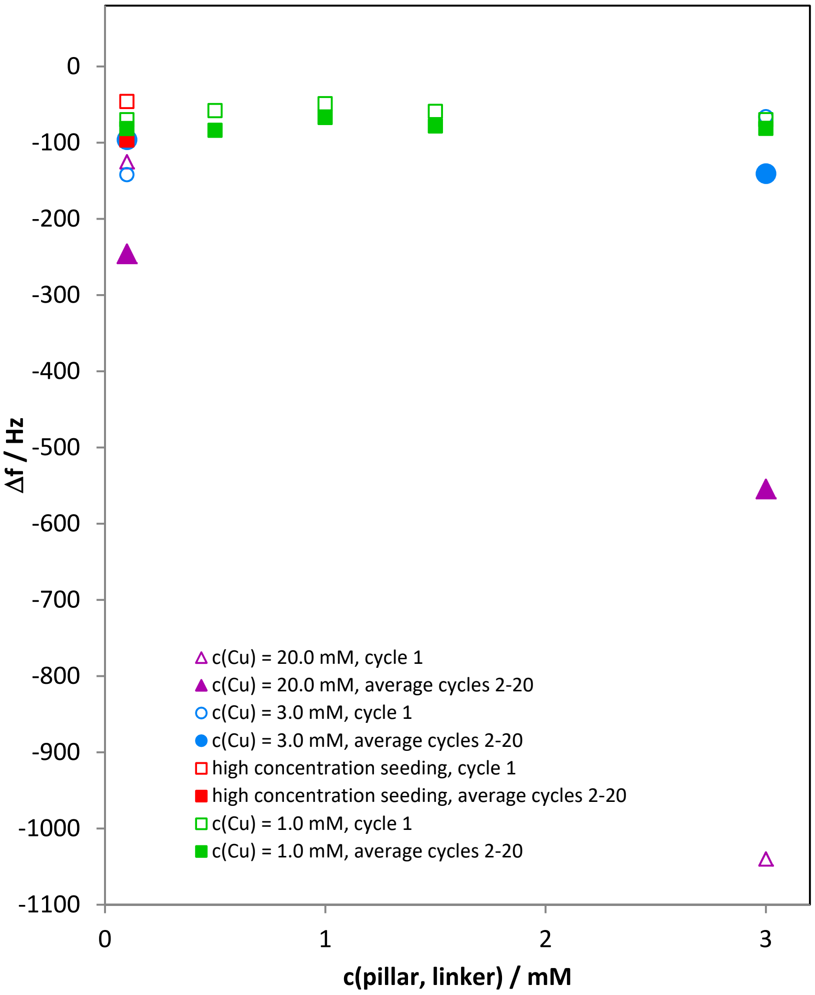

Figure S39. Relative mass changes, given by frequency changes Δf, upon rinsing with pillar/linker solution and subsequent washing with pure ethanol in dependence on the Cu^{2+} and pillar/linker solution concentrations. "High concentration seeding": 1st cycle with $c_{Cu^{2+}}$ = 1 mM, c(pillar, linker) = 3.0 mM and cycles 2-20: $c_{Cu^{2+}}$ = 1 mM, c(pillar, linker) = 0.1 mM.

Orientation of the SURMOFs

Proportion of (001) Orientation

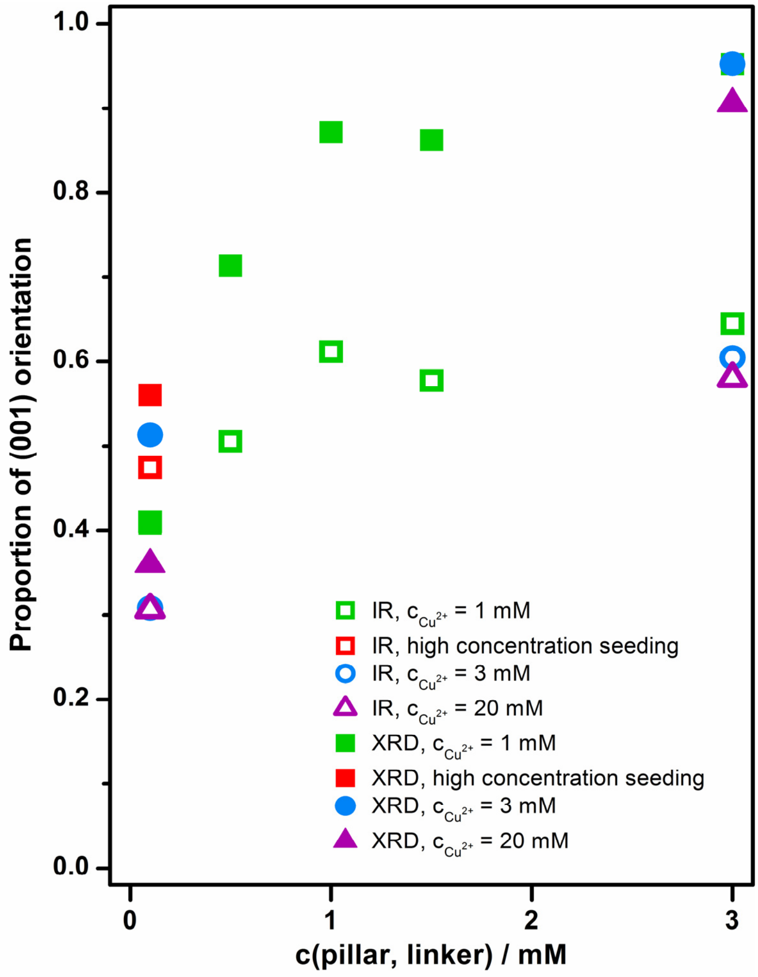

Figure S40. Proportion of (001) oriented paddlewheel units in the $Cu_2(F_4bdc)_2(dabco)$ SURMOFs on PPP1@Au in dependence of the concentrations of Cu^{2+} and pillar/linker solutions. Data were obtained using the formula derived in ref.[3] Note that this derivation is applicable only if all paddlewheels in the SURMOF are either oriented (001) or perpendicular to (001). If the values obtained from IR and from XRD deviate for a given sample, this is interpreted as an indication of the occurrence of crystallites with other orientations. "High concentration seeding": 1st cycle with $c_{Cu^{2+}}$ = 1 mM, c(pillar, linker) = 3.0 mM and cycles 2-20: $c_{Cu^{2+}}$ = 1 mM, c(pillar, linker) = 0.1 mM.

Average tilt angle from IR data

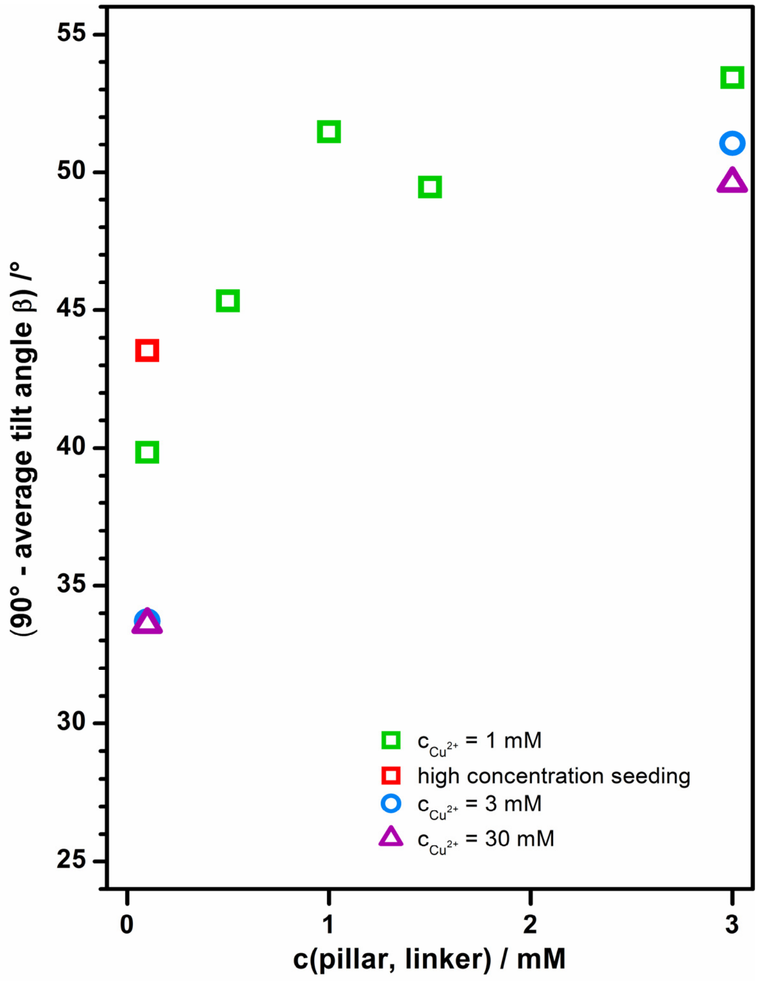

Figure S41. Average tilt of the paddlewheel units in the SURMOFs with respect to the surface (i.e., 90° - β) in dependence of the concentrations of Cu^{2+} and pillar/linker solutions. For derivation of the formula to obtain β, *vide infra*. "High concentration seeding": 1st cycle with $c_{Cu^{2+}}$ = 1 mM, c(pillar, linker) = 3.0 mM and cycles 2-20: $c_{Cu^{2+}}$ = 1 mM, c(pillar, linker) = 0.1 mM.

Surface density and sizes of SURMOF crystallites

Surface density

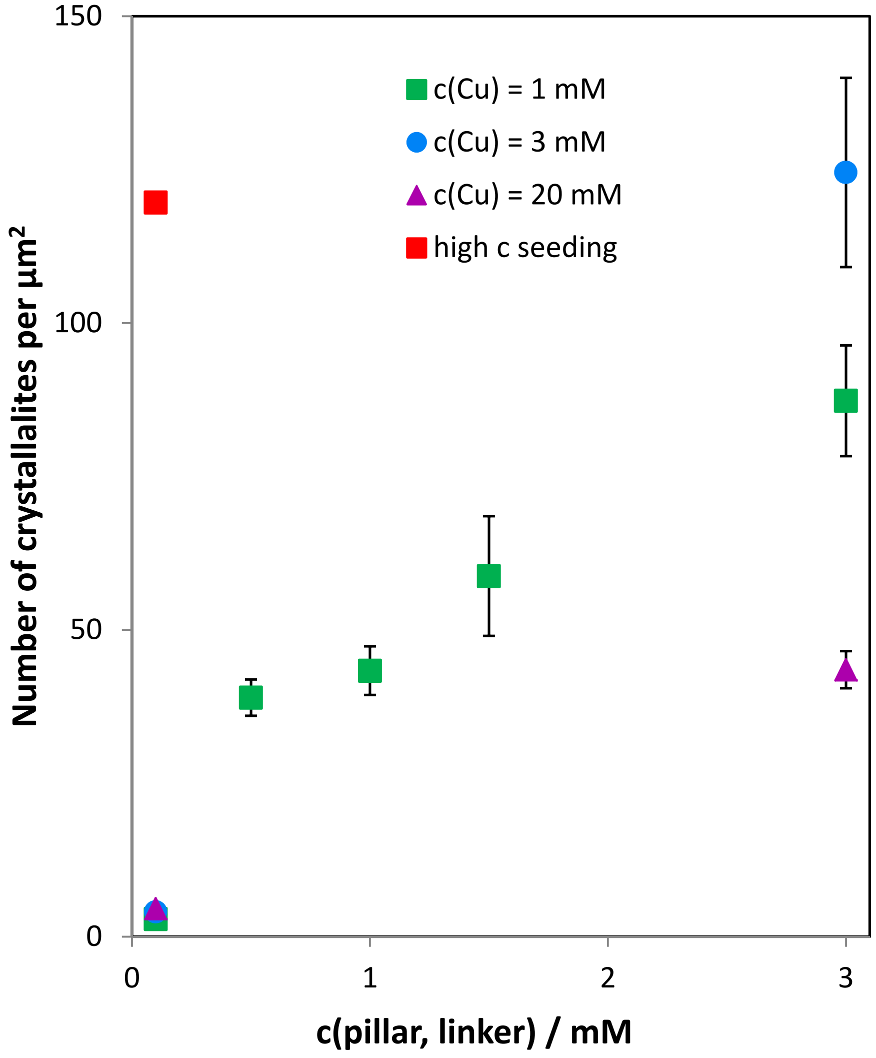

Figure S42. Number of crystallites per μm^2 in the SURMOFs in dependence of pillar, linker- and copper concentrations, determined by evaluation of SEM images. "High concentration seeding": 1st cycle with $c_{Cu^{2+}}$ = 1 mM, c(pillar, linker) = 3.0 mM and cycles 2-20: $c_{Cu^{2+}}$ = 1 mM, c(pillar, linker) = 0.1 mM.

Approximate crystallite sizes

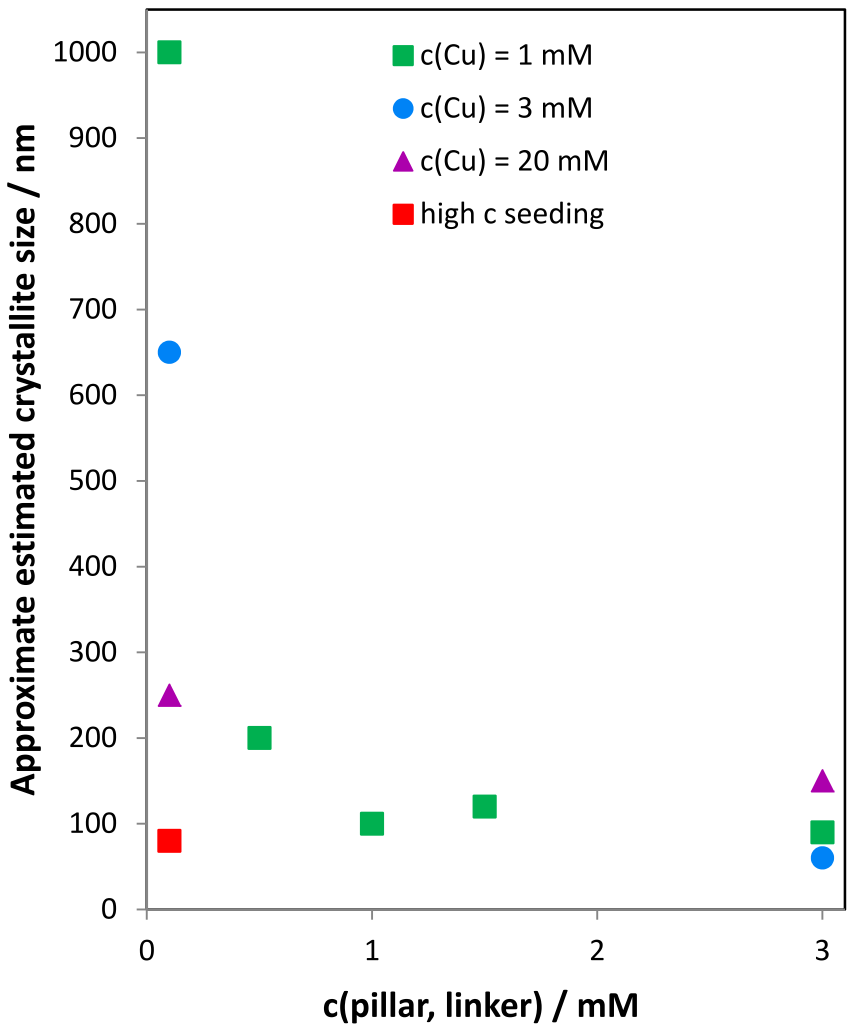

Figure S43. Approximate crystallite sizes in the SURMOFs in dependence of pillar, linker- and copper concentrations, determined by evaluation of SEM images . "High concentration seeding": 1st cycle with $c_{Cu^{2+}}$ = 1 mM, c(pillar, linker) = 3.0 mM and cycles 2-20: $c_{Cu^{2+}}$ = 1 mM, c(pillar, linker) = 0.1 mM. Note that depending on the preparation conditions, the shape of the crystallites and their size distribution undergo significant changes, which hampers the comparability of the plotted numbers. These data should therefore be taken rather qualitatively. General trends are apparent anyway: (i) the higher c(pillar, linker), the lower the crystallite size, and (ii) the higher c(Cu), the lower the crystallite size. High c seeding represents an exception (small crystallites at low concentrations).

Roughness of SURMOFs

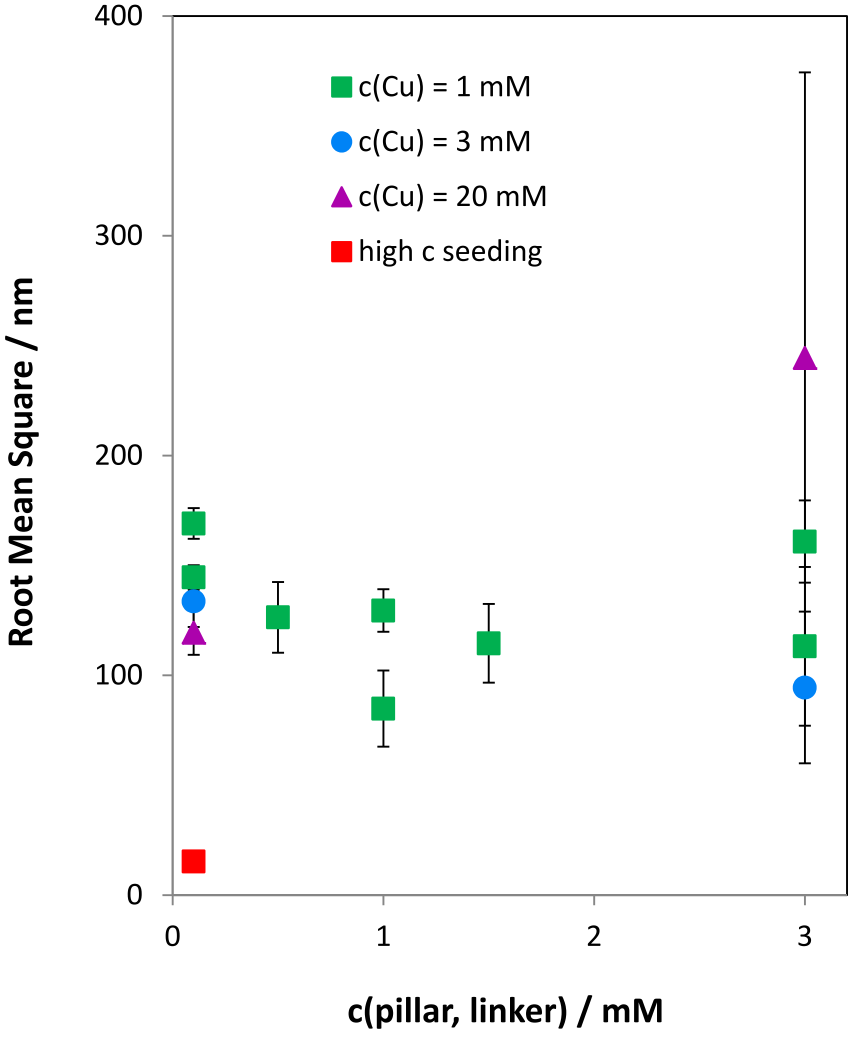

Figure S44. Root mean square roughness of SURMOFs in dependence of pillar, linker- and copper concentrations, determined by AFM. "High concentration seeding": 1st cycle with $c_{Cu^{2+}}$ = 1 mM, c(pillar, linker) = 3.0 mM and cycles 2-20: $c_{Cu^{2+}}$ = 1 mM, c(pillar, linker) = 0.1 mM.

LbL experiment with increased durations of pumping cycles

Following a suggestion during the review process, an additional experiment with increased durations of the pumping cycles (800 s copper(II) acetate solution, 400 seconds absolute ethanol, 4000 seconds $H_2(F_4bdc)$/dabco solution, 800 seconds absolute ethanol) was performed at low concentrations, namely $c(Cu^{2+})$ = 1.0 mM, c(dabco) = 0.1 mM and $c(H_2F_4bdc)$ = 0.1 mM, to check whether full coverage can be achieved under these conditions. Note that this LbL experiment takes twice as long as the other ones.

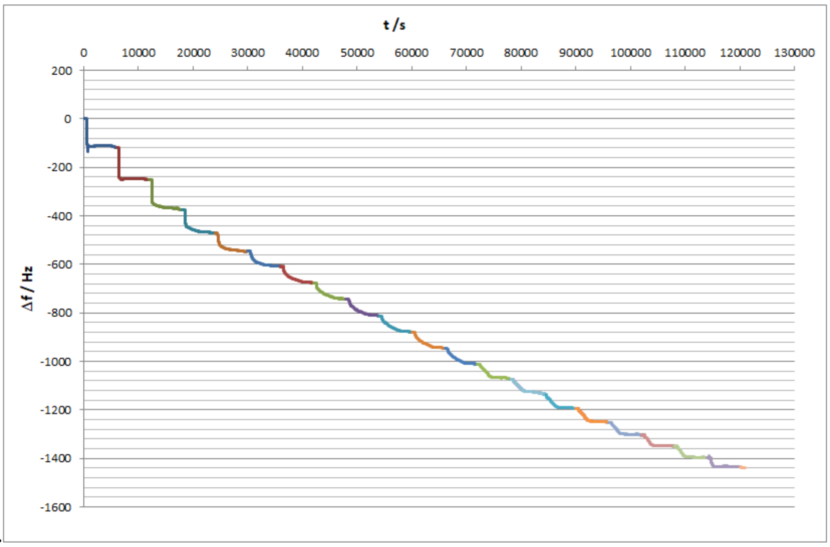

Figure S45. QCM curve of the LbL experiment with $c(Cu^{2+})$ = 1.0 mM, c(dabco) = 0.1 mM and $c(H_2F_4bdc)$ = 0.1 mM at increased duration of pumping cycles.

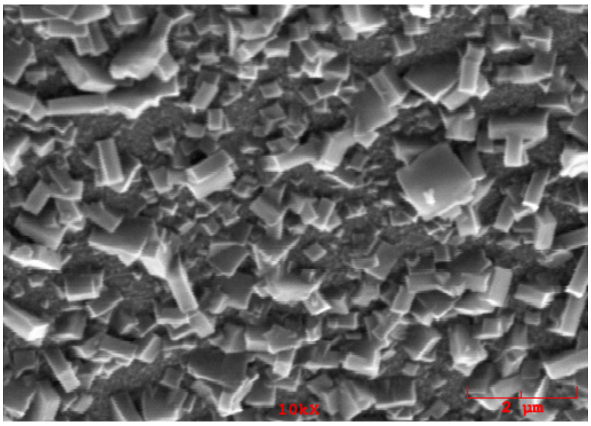

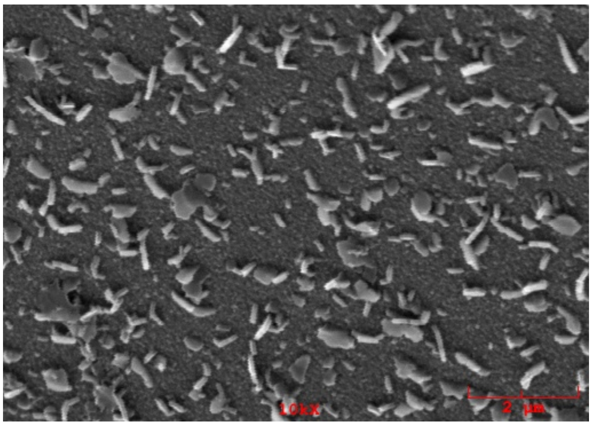

Figure S46. SEM images of the substrate after the LbL experiment with $c(Cu^{2+})$ = 1.0 mM, c(dabco) = 0.1 mM and $c(H_2F_4bdc)$ = 0.1 mM at increased duration of pumping cycles.

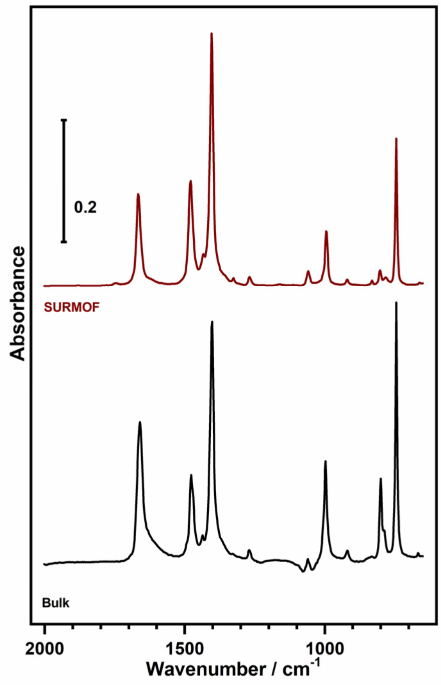

Figure S47. IR spectrum of of the substrate after the LbL experiment with c(Cu^{2+}) = 1.0 mM, c(dabco) = 0.1 mM and c(H_2F_4bdc) = 0.1 mM at increased duration of pumping cycles (upper trace) along with a bulk spectrum of $Cu_2(F_4bdc)_2(dabco)$ (lower trace, with signal strength in arbitrary units).

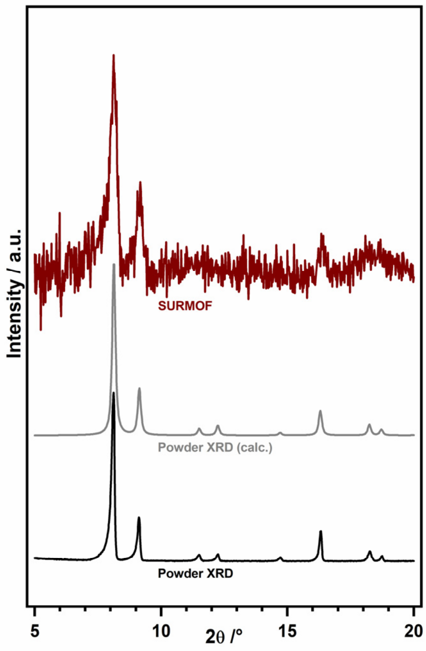

Figure S48. XRD pattern of the substrate after the LbL experiment with c(Cu^{2+}) = 1.0 mM, c(dabco) = 0.1 mM and c(H_2F_4bdc) = 0.1 mM at increased duration of pumping cycles (upper trace) along with an experimental and a calculated powder MOF diffractogram of $Cu_2(F_4bdc)_2(dabco)$.

Discussion of the results: An at first sight surprisingly low amount of material was deposited compared to most of the other SURMOFs (only slightly more than in case of the high concentration seeding - see Fig. S21, and comparable to the low concentration experiment - see Fig. S3). IR and XRD are in accord with the assumption that the desired SURMOF has formed. SEM reveals a somewhat inhomogeneous distribution of crystallites of sizes similar and smaller than in case of the low concentration LbL experiment (compare with Fig. S23). A full coverage could not be achieved.

Evaluation Formula to Obtain the Average Tilt Angle from IR Intensities

In this section, a formula is derived that relates the relative absorbance signal strengths A_s and A_{as} of the symmetric (s) and asymmetric (as) carboxylate stretch modes in the SURMOF and bulk MOF IR spectra with an average tilt angle β of the paddlewheel units in the SURMOF. The quotients Q = A_{as}/A_s in the bulk MOF spectrum and in a SURMOF spectrum will be different since in the bulk material, the paddlewheels are uniformly distributed, while in the SURMOF, an alignment of the paddlewheel axes is assumed. Such alignment alters the IR signal strength because of the surface selection rule for metals,[8] which states that only the component of the transition dipole moment parallel to the surface normal can contribute to the detectable signal.

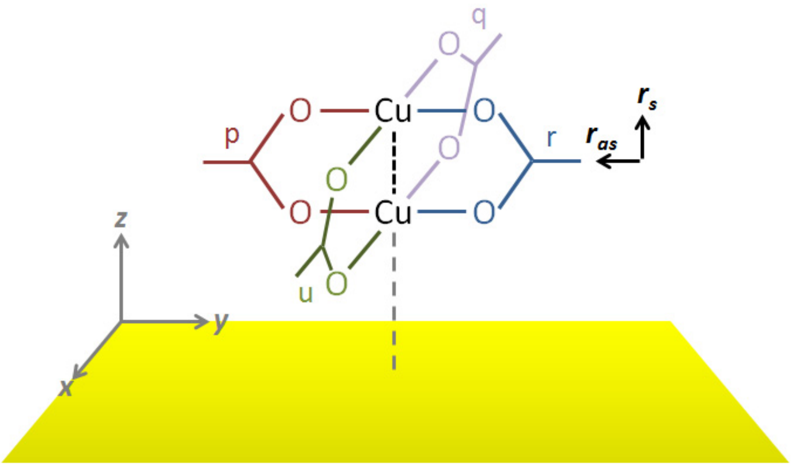

Figure S49. Sketch to illustrate the meaning of various directions in the SURMOF. In the Cu paddlewheel motif, four carboxylate moieties p, q, r, and u are present, each of which contributes to the absorbance signal strengths of the symmetric and asymmetric carboxyl stretch vibration bands in the IRRA spectrum of the SURMOF. In a hypothetical starting orientation, the main axis of the paddlewheel (black dashed line that connects the Cu atoms) is parallel to the substrate surface normal **z** and by definition, the tilt angle β of the paddlewheel then is zero. Exemplary, the transition dipole moment vectors $\boldsymbol{r_s}$ = $(0,0,t_s)$ and $\boldsymbol{r_{as}}$ = $(0,-t_{as},0)$ that belong to the moiety r are given in the figure (black arrows). By subsequent rotation about the $\boldsymbol{z}$ axis and the $\boldsymbol{x}$ axis, the paddlewheel and all transition dipole moment vectors are transferred into the orientations that are supposed to be existent in the SURMOF.

We start by defining the transition dipole moment vectors of all carboxyl groups of one paddlewheel unit as present in the $Cu_2(F_4bdc)_2(dabco)$ MOF structure. Its initial (hypothetical) orientation is upright, i.e., the axis between the two Cu atoms in the paddlewheel is parallel to the substrate surface normal $\boldsymbol{z}$ = (0,0,1) and the tilt angle β, defined as the angle between the Cu - Cu axis and the surface normal, is zero. Note that the Cu - Cu axis represents the crystallographic direction (001) of the $Cu_2(F_4bdc)_2(dabco)$ MOF. When the paddlewheel is oriented with a tilt of β = 0°, the transition dipole moment vectors related to the carboxyl groups displayed in Fig. S49 are represented by:

$$\boldsymbol{p}_{as} = \begin{pmatrix} 0 \\ 0 \\ t_{as} \end{pmatrix}, \quad \boldsymbol{q}_{as} = \begin{pmatrix} 0 \\ 0 \\ t_{as} \end{pmatrix}, \quad \boldsymbol{r}_{as} = \begin{pmatrix} 0 \\ 0 \\ t_{as} \end{pmatrix}, \quad \boldsymbol{u}_{as} = \begin{pmatrix} 0 \\ 0 \\ t_{as} \end{pmatrix},$$

$$\boldsymbol{p}_{s} = \begin{pmatrix} 0 \\ -t_s \\ 0 \end{pmatrix}, \quad \boldsymbol{q}_{s} = \begin{pmatrix} -t_s \\ 0 \\ 0 \end{pmatrix}, \quad \boldsymbol{r}_{s} = \begin{pmatrix} 0 \\ t_s \\ 0 \end{pmatrix}, \quad \boldsymbol{u}_{s} = \begin{pmatrix} t_s \\ 0 \\ 0 \end{pmatrix}. \tag{S1}$$

with t_{as} and t_s = the absolute values of the asymmetric and symmetric transition dipole moments, respectively. The main molecular axis is (0,0,1).

To transfer them into their final orientations, the paddlewheels and the transition dipole moments are in a first step rotated about the z-axis by the angle γ. The matrix representation of this operation is:

$$\underline{R_z}(\gamma) = \begin{pmatrix} \cos\gamma & -\sin\gamma & 0 \\ \sin\gamma & \cos\gamma & 0 \\ 0 & 0 & 1 \end{pmatrix}$$

and, for example, the application of this matrix to $\boldsymbol{p}_{as}$ results in:

$$\boldsymbol{p'}_{as} = \underline{R_z}(\gamma)\,\boldsymbol{p}_{as} = \begin{pmatrix} \cos\gamma & -\sin\gamma & 0 \\ \sin\gamma & \cos\gamma & 0 \\ 0 & 0 & 1 \end{pmatrix}\begin{pmatrix} 0 \\ 0 \\ t_{as} \end{pmatrix} = \begin{pmatrix} 0 \\ 0 \\ t_{as} \end{pmatrix} \tag{S2}$$

After rotation about z by the γ, the transition dipole moment vectors are:

$$\boldsymbol{p'}_{as} = t_{as}\cdot\begin{pmatrix} 0 \\ 0 \\ 1 \end{pmatrix}, \quad \boldsymbol{q'}_{as} = t_{as}\cdot\begin{pmatrix} 0 \\ 0 \\ 1 \end{pmatrix}, \quad \boldsymbol{r'}_{as} = t_{as}\cdot\begin{pmatrix} 0 \\ 0 \\ 1 \end{pmatrix}, \quad \boldsymbol{u'}_{as} = t_{as}\cdot\begin{pmatrix} 0 \\ 0 \\ 1 \end{pmatrix},$$

$$\boldsymbol{p'}_{s} = t_s\cdot\begin{pmatrix} \sin\gamma \\ -\cos\gamma \\ 0 \end{pmatrix}, \quad \boldsymbol{q'}_{s} = t_s\cdot\begin{pmatrix} -\cos\gamma \\ -\sin\gamma \\ 0 \end{pmatrix}, \quad \boldsymbol{r'}_{s} = t_s\cdot\begin{pmatrix} -\sin\gamma \\ \cos\gamma \\ 0 \end{pmatrix}, \quad \boldsymbol{u'}_{s} = t_s\cdot\begin{pmatrix} \sin\gamma \\ \cos\gamma \\ 0 \end{pmatrix}, \tag{S3}$$

and the main molecular axis vector still is (0,0,1).

In the final step, the paddlewheel and the transition dipole moments are rotated about the $\boldsymbol{x}$-axis by the angle β. The matrix representation of this rotational operation is:

$$\underline{R_x}(\beta) = \begin{pmatrix} 1 & 0 & 0 \\ 0 & \cos\beta & -\sin\beta \\ 0 & \sin\beta & \cos\beta \end{pmatrix}$$

and, for example, the application of this matrix to $\boldsymbol{p'}_{as}$ results in:

$$\boldsymbol{p''}_{as} = \underline{R_x}(\beta)\,\boldsymbol{p'}_{as} = \begin{pmatrix} 1 & 0 & 0 \\ 0 & \cos\beta & -\sin\beta \\ 0 & \sin\beta & \cos\beta \end{pmatrix}\begin{pmatrix} 0 \\ 0 \\ t_{as} \end{pmatrix} = t_{as}\cdot\begin{pmatrix} 0 \\ -\sin\beta \\ \cos\beta \end{pmatrix}. \tag{S4}$$

After rotation about $\boldsymbol{x}$ by the angle β, the transition dipole moment vectors are:

$$\boldsymbol{p''_{as}} = t_{as} \cdot \begin{pmatrix} 0 \\ -\sin\beta \\ \cos\beta \end{pmatrix}, \qquad \boldsymbol{q''_{as}} = t_{as} \cdot \begin{pmatrix} 0 \\ -\sin\beta \\ \cos\beta \end{pmatrix},$$

$$\boldsymbol{r''_{as}} = t_{as} \cdot \begin{pmatrix} 0 \\ -\sin\beta \\ \cos\beta \end{pmatrix}, \qquad \boldsymbol{u''_{as}} = t_{as} \cdot \begin{pmatrix} 0 \\ -\sin\beta \\ \cos\beta \end{pmatrix},$$

$$\boldsymbol{p''_{s}} = t_{s} \cdot \begin{pmatrix} \sin\gamma \\ -\cos\beta\cos\gamma \\ -\sin\beta\cos\gamma \end{pmatrix}, \qquad \boldsymbol{q''_{s}} = t_{s} \cdot \begin{pmatrix} -\cos\gamma \\ -\cos\beta\sin\gamma \\ -\sin\beta\sin\gamma \end{pmatrix}, \qquad \text{(S5)}$$

$$\boldsymbol{r''_{s}} = t_{s} \cdot \begin{pmatrix} -\sin\gamma \\ \cos\beta\cos\gamma \\ \sin\beta\cos\gamma \end{pmatrix}, \qquad \boldsymbol{u''_{s}} = t_{s} \cdot \begin{pmatrix} \cos\gamma \\ \cos\beta\sin\gamma \\ \sin\beta\sin\gamma \end{pmatrix},$$

and the main molecular axis vector now is (0, -sin β, cos β), i.e. the paddlewheel unit now has a tilt angle of β. For this tilted paddlewheel, the IRRAS absorbance signals strengths A_s and A_{as} of the symmetric and asymmetric carboxylate stretch vibration modes need to be calculated. These quantities are proportional to the square of the scalar products of their transition dipole moment vectors and the e-field vector of the impinging IR radiation $\boldsymbol{e}$ = (0, sin ε, cos ε). The angle of irradiation relative to the substrate surface normal ε = 80° in the experiments conducted in this study, yet this plays no specific role in the current derivation. It is of much more importance to note that only the z-components of all transition dipole moment vectors need to be considered if the surface selection rule on metals is valid.[8] This results the following expressions for absorbance signal strengths for the IR spectra of the SURMOFs:

$$\begin{aligned} A_{as}(\text{SURMOF}) &= [z(\boldsymbol{p''_{as}}) \cdot z(\boldsymbol{e})]^2 + [z(\boldsymbol{q''_{as}}) \cdot z(\boldsymbol{e})]^2 + [z(\boldsymbol{r''_{as}}) \cdot z(\boldsymbol{e})]^2 + [z(\boldsymbol{u''_{as}}) \cdot z(\boldsymbol{e})]^2 \\ &= t_{as}^2 \cdot \cos^2\beta \cdot \cos^2\varepsilon + t_{as}^2 \cdot \cos^2\beta \cdot \cos^2\varepsilon + t_{as}^2 \cdot \cos^2\beta \cdot \cos^2\varepsilon + t_{as}^2 \cdot \cos^2\beta \cdot \cos^2\varepsilon \qquad \text{(S6)} \\ &= 4 \cdot t_{as}^2 \cdot \cos^2\beta \cdot \cos^2\varepsilon \end{aligned}$$

and

$$\begin{aligned} A_{s}(\text{SURMOF}) &= [z(\boldsymbol{p''_{s}}) \cdot z(\boldsymbol{e})]^2 + [z(\boldsymbol{q''_{s}}) \cdot z(\boldsymbol{e})]^2 + [z(\boldsymbol{r''_{s}}) \cdot z(\boldsymbol{e})]^2 + [z(\boldsymbol{u''_{s}}) \cdot z(\boldsymbol{e})]^2 \\ &= t_s^2 \cdot \sin^2\beta \cdot \cos^2\gamma \cdot \cos^2\varepsilon + t_s^2 \cdot \sin^2\beta \cdot \sin^2\gamma \cdot \cos^2\varepsilon + t_s^2 \cdot \sin^2\beta \cdot \cos^2\gamma \cdot \cos^2\varepsilon \\ &\qquad + t_s^2 \cdot \sin^2\beta \cdot \sin^2\gamma \cdot \cos^2\varepsilon \qquad \text{(S7)} \\ &= t_s^2 \cdot \cos^2\varepsilon \cdot \sin^2\beta \cdot (\cos^2\gamma + \sin^2\gamma + \cos^2\gamma + \sin^2\gamma) \\ &= t_s^2 \cdot \cos^2\varepsilon \cdot \sin^2\beta \cdot 2 \end{aligned}$$

We define the ratio of the signal strengths of the s and as carboxylate bands in one IR spectrum of a SURMOF as:

$$Q_{SURMOF} = \frac{A_{as}(SURMOF)}{A_s(SURMOF)} = \frac{4 \cdot t_{as}^2 \cdot \cos^2\beta \cdot \cos^2\varepsilon}{2 \cdot t_s^2 \cdot \cos^2\varepsilon \cdot \sin^2\beta} = \frac{t_{as}^2}{t_s^2} \cdot \frac{2}{\tan^2\beta} \tag{S8}$$

Since in the bulk MOF, the orientations of the paddlewheel units can be considered to be uniformly distributed, the ratio of absorption signal strengths in the bulk MOF spectrum is

$$Q_{bulk} = \frac{A_{as}(bulk)}{A_s(bulk)} = \frac{t_{as}^2}{t_s^2} \tag{S9}$$

Both Q_{SURMOF} and Q_{bulk} are experimentally accessible by evaluation of the IR spectra of the SURMOF and the bulk MOF. Hence, the average tilt angle of the paddlewheel moieties in the SURMOF can be obtained by combining equations S8 and S9, which leads to the formula

$$\beta = \tan^{-1}\left(\sqrt{\frac{2 \cdot Q_{bulk}}{Q_{SURMOF}}}\right) \quad . \tag{S10}$$

References

[1] R. Kitaura, F. Iwahori, R. Matsuda, S. Kitagawa, Y. Kubota, M. Takata, T. C. Kobayashi, *Inorganic Chemistry* **2004**, *43*, 6522–6524.
[2] B. Schüpbach, A. Terfort, *Organic & Biomolecular Chemistry* **2010**, *8*, 3552.
[3] J.-L. Zhuang, M. Kind, C. M. Grytz, F. Farr, M. Diefenbach, S. Tussupbayev, M. C. Holthausen, A. Terfort, *Journal of the American Chemical Society* **2015**, *137*, 8237–8243.
[4] M. J. Frisch, G. W. Trucks, H. B. Schlegel, G. E. Scuseria, M. A. Robb, J. R. Cheeseman, J. A. Montgomery Jr., T. Vreven, K. N. Kudin, J. C. Burant, et al., *Gaussian 09, Revision A.02*, Gaussian, Inc., Wallingford, CT, **2004**.
[5] J. P. Perdew, *Physical Review B* **1986**, *33*, 8822–8824.
[6] A. D. Becke, *Physical review A* **1988**, *38*, 3098–3100.
[7] F. Weigend, R. Ahlrichs, *Physical Chemistry Chemical Physics* **2005**, *7*, 3297.
[8] R. G. Greenler, *The Journal of Chemical Physics* **1966**, *44*, 310–315.
